# Non-local synchronization of continuous time crystals in a semiconductor

**DOI:** 10.1038/s41467-026-75714-1

**Published:** 2026-07-22

**Authors:** Alex Greilich, Nataliia E. Kopteva, Vladimir L. Korenev, Philipp A. Haude, Linus Kunze, Ben W. Grobecker, Sergiu Anghel, Markus Betz, Manfred Bayer

**Affiliations:** Experimentelle Physik 2, Technische Universität, Dortmund, Germany

**Keywords:** Semiconductors, Nonlinear phenomena

## Abstract

Synchronization resulting in unified collective behavior of the individual elements of a system that are weakly coupled to each other has long fascinated scientists. Examples range from the periodic oscillation of coupled pendulum clocks to the rhythmic behavior in biological systems. Here we demonstrate this effect in a solid-state platform: spatially remote, auto-oscillating electron-nuclear spin systems in a semiconductor. When two such oscillators separated by up to 40 *μ*m are optically pumped, their individually different frequencies lock to a common value, revealing long-range coupling. For larger separations, the synchronization breaks. The interaction distance matches the electron spin diffusion length, identifying spin transport as the coupling-mediating mechanism and maintaining correlated behavior over mesoscopic distances. As a consequence, a wide-area optical pump drives all oscillators within the illuminated spot into a single synchronized state, despite their inhomogeneity. This synchronization accounts for the exceptional stability of the resulting auto-oscillations, enabling collective motion in distributed spin systems and paving the way toward spin networks in spintronics.

## Abstract (web summary)

Synchronization of weakly coupled oscillators is a widespread phenomenon underlying collective behavior in physical and biological systems. Here, the authors demonstrate mutual synchronization of auto oscillating electron–nuclear spin ensembles in a semiconductor, showing frequency locking over tens of micrometers mediated by electron spin diffusion.

## Introduction

Synchronization is a universal phenomenon in which auto-oscillators adjust their rhythms to operate in unison. The defining feature is that auto-oscillators, capable of developing periodic motion, adjust their frequencies through weak interactions while retaining their individuality. Synchronization can arise mutually, when oscillators interact with each other, or be driven by an external periodic force^1,2^. Huygens’ 17th-century observation that two pendulum clocks on the same beam swing in phase demonstrated how mechanical systems can couple through a common medium to lock their motion^3,4^. Since then, synchronization has been recognized as a general principle across living and non-living systems.

In biology, it underlies fireflies flashing in concert^5^, cardiac pacemaker cells beating in harmony^6^, and circadian clocks aligning to day-night cycles^7,8^. In neuroscience, populations of neurons coordinate oscillatory firing to produce brain rhythms^9^. In the physical sciences, synchronization is central to micro- and nano-scale devices: spin-torque nano-oscillators can phase-lock via electrical coupling, enhancing signal power and spectral purity^10–12^. Josephson junctions^13^, laser arrays^14^, and optomechanical oscillators^15^ similarly exhibit mutual entrainment, enabling coherent operation of many units. In atomic physics, synchronization occurs in driven ensembles where global coupling can overcome dephasing. As an example, Wadenpfuhl et al.^16^ showed that thermal rubidium atoms excited to Rydberg states exhibit synchronized oscillations due to mean-field interactions via a shared optical field.

Auto-oscillating systems produce narrow Fourier peaks in the frequency domain, providing an analogy to spatial crystals. So, in crystallography, sharp peaks in momentum space, known as Bragg peaks, arise from constructive interference of waves scattered by atomic planes, reflecting the underlying periodic lattice structure in space^17^. By this analogy, we refer to an auto-oscillator that exhibits spontaneous, persistent oscillations breaking the continuous time-translation symmetry as a time crystal^18,19^. A synchronized ensemble of such auto-oscillators can then also be regarded as a time crystal composed of many unit cells. These systems can be divided into discrete-time crystals, which are non-autonomous systems responding at subharmonic frequencies under periodic driving^20,21^, and continuous-time crystals (CTCs), which are autonomous systems sustained by constant driving and exhibiting self-oscillations. The latter have been realized in many-body systems with long-range interactions, such as Magnon Bose-Einstein condensates^22^, Rubidium Bose-Einstein condensates^23^, photonic nanomaterials^24,25^, Rydberg gas systems^26^, and polariton condensates in semiconductors^27^.

Electron-nuclear spin systems in InGaAs semiconductors can also exhibit time crystal dynamics. Recent work has demonstrated robust auto-oscillations in coupled electron and nuclear spin polarizations under continuous circularly polarized excitation, thereby realizing a continuous time crystal^28^. Furthermore, these oscillations can synchronize with an externally applied modulation when its frequency approaches their natural resonance^29^, also showing a subharmonic response. Here, we demonstrate mutual synchronization between autonomous oscillators in a semiconductor system that persists even when their natural frequencies differ by up to 40%. Distinct spin ensembles, comprising up to 10^9^ auto-oscillators, spontaneously lock to a common rhythm, revealing the emergence of a robust collective phase. The key new elements of the present work are the demonstration of spatial synchronization, the observation of long-range coupling between separated spin ensembles, and the identification of spin diffusion as the underlying coupling mechanism, which is further corroborated by theoretical modeling. These results open new pathways toward building spin networks and exploring spatially extended regimes of macroscopic synchronization.

## Results

### Auto-oscillations

The first step of our study is to establish robust auto-oscillations of the electron-nuclear spin system in 10 μm thick InGaAs epilayer with 3% indium content. It is homogeneously doped with Si atoms, with a dopant density of *n*_*e*_ = 39 × 10^15^ cm^−3^^30^. Localization of electrons at the Si-donors leads to an enhanced hyperfine interaction with the nuclei, in contrast to free electrons. Localized electrons, oriented by circularly polarized pump light, transfer their spin polarization to the surrounding nuclei through the hyperfine interaction. The resulting nuclear polarization generates an Overhauser field that feeds back on the electron spins. This allows one to monitor nuclear spin dynamics via the time evolution of the electron spin polarization, as measured by the Faraday rotation of the linearly polarized probe laser. Being an open system, the nuclei continuously dissipate energy, yet this loss can be compensated by optical pumping of the donor-bound electron spins^28^. Under appropriate sample design and experimental conditions, this feedback loop generates non-decaying auto-oscillations in the electron-nuclear spin system (ENSS) - a realization of the CTC^28^. The main experimental parameters and sample features are detailed in the Methods and the Supplementary section 1.

**Fig. 1:**
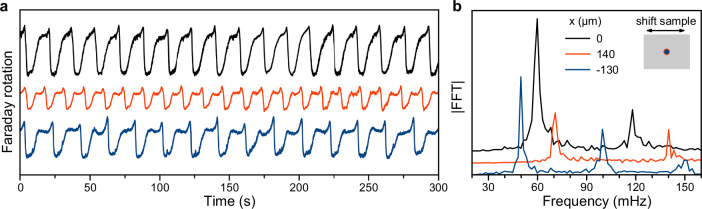
Inhomogeneity. **a** Examples of time traces measured at different sample positions. Pump and probe spots are overlapped, and the sample is shifted in the range of  − 150 μm to  + 150 μm along the horizontal direction (x). Pump spot diameter at 1/*e*^2^ intensity level is equal to 22 μm (FWHM of 13 μm). Probe spot size at 1/*e*^2^ = 12 μm (FWHM of 7 μm). Pump/Probe powers are 0.15/1 mW, respectively. **b** Corresponding fast Fourier transform spectra of the traces from (**a**) measured for 10 min at the arbitrary position x = 0, the top black trace; to the right side of the previous position at x = 140 μm, red trace; and to the left side at x = − 130 μm, blue trace. The difference in frequencies is related to the intrinsic sample variation.

Each donor electron, coupling to ~10^6^ nuclear spins within its wavefunction characterized by the Bohr radius of 11 nm^30^, defines the volume of a single auto-oscillator. In effect, the situation is better described by a flux of free electrons through the donor that collectively polarize the nuclear spin ensemble, so that the experimentally accessible quantity is the average electron spin polarization at the donor. Finally, the intrinsic variation of the semiconductor sample, related to fluctuations in donor density and indium incorporation, results in variations of the observed oscillation frequencies as shown in Fig. 1. Here, the pump and probe spots are tightly focused (see figure caption) and overlapped, while the sample is shifted in the horizontal direction by up to ±150 μm. Figure 1a demonstrates exemplary time traces for three different positions on the sample. The fast Fourier transforms (FFT) for the 10-minute time traces are shown in Fig. 1b, with the measurement positions marked in the panel. The frequency variation is pronounced. This feature is a significant advantage of our system, owing to the systematic variation in the auto-oscillation frequency across the sample.

To highlight, the number of participating donors in the pump spot focused to a size of 22 *μ*m, is on the order of ${{\mathcal{O}}}(1{0}^{7})$. Despite that, the observed linewidth of the FFT is determined by the inverse measurement time of the non-decaying oscillations, without any dephasing that one could expect for a large number of participating oscillators. This raises the question of whether all the individual auto-oscillators, each defined by a single donor, synchronize and give rise to a macroscopic collective behavior?

### Single-pump mutual synchronization

**Fig. 2:**
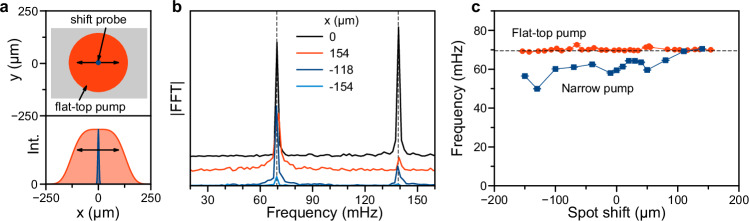
Mutual synchronization. **a** Sketch of experiment: red pump spot is prepared by a *π*Shaper to produce a flat-top intensity distribution on the sample, as seen in the simulated bottom part of the figure. Pump spot diameter is about 200 μm. The Gaussian probe beam is focused down to 12 μm at 1/*e*^2^ (FWHM of 7 μm) and can be shifted horizontally within the pump beam by the same distances as in the case of Fig. 1a. The bottom part shows the simulated profiles of the lasers. Pump/Probe powers  = 10/1 mW. **b** Examples of the fast Fourier transform spectra of the traces measured for 10 min at the center x = 0, the top black trace; on the right side of the pump center at x = 154 μm, red trace; and on the left side at x = − 118 μm, blue trace, and at x = − 154 μm, light blue trace. The difference in frequencies is now negligible compared to Fig. 1b, suggesting complete synchronization within the pump-spot excitation. **c** Summarized data for the FFT peak positions of the first harmonics for both experimental cases versus shift of the sample for tightly focused pump (blue squares) or the probe shift within a single widened pump (red circles).

To answer the question of whether different auto-oscillations synchronize within a single pump spot, we designed an experiment in which we fixed the sample position and shifted the tightly focused probe spot (12 μm) within the broadened pump (200 μm) for the same range of sample positions as in the previous setting. The pump spot was prepared in a flat-top configuration to provide similar excitation conditions within the excitation spot diameter, as shown in Fig. 2a. Figure 2b again demonstrates exemplary FFTs for several probe positions inside the pump spot, showing that all observed frequencies across the pump spot are now equal. Figure 2c reflects the difference in the observed FFT frequencies of the first harmonic for both cases: blue squares demonstrate the variation of the frequency over the sample measured with a tightly focused pump spot, while the red circles show that all frequencies are now equalized and are independent of the probe spot position within the single widened pump. These results can be interpreted as a clear demonstration of the mutual synchronization of spatially separated oscillators in an excited ensemble of auto-oscillators. It is essential to note that the relative deviation in frequencies synchronized within a single pump can reach up to 40%. The number of the involved donors in that case can be estimated to be about ${{\mathcal{O}}}(1{0}^{9})$. Importantly, the present measurements of synchronization within a single pump spot clarify the remarkable robustness of the auto-oscillations reported previously^28^: their stability arises from the emergence of a many-body synchronized state, which protects the dynamics against fluctuations of external parameters. This last demonstration is also well captured by a simplified model in which a single auto-oscillator is used to describe the whole system^28,29,31^.

### Two-pump synchronization

**Fig. 3:**
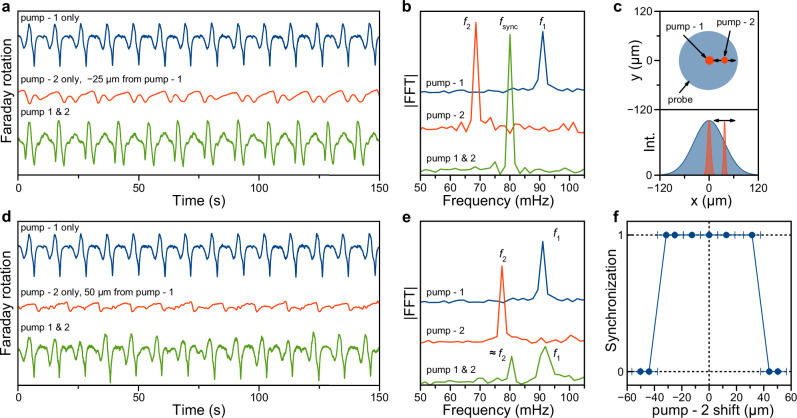
Range of synchronization. **a** Experimental time traces of auto-oscillations for three different cases: the top blue one shows the pump-1 only, the red one in the middle is the pump-2 only, shifted horizontally by  − 25 μm away from the pump-1 position. Finally, the last green time trace at the bottom represents the combined application of pump-1 and pump-2. See Fig. 3 c for the labeling of the pumps. **b** First harmonic range of the fast Fourier transform spectrum of the traces presented in (**a**), measured for 10 min. As one can see, each separate pump beam at its sample position induces auto-oscillations with a characteristic frequency. If both pumps are applied together, one can see a single frequency, a hallmark of synchronization. Laser powers for Pump-1/Pump-2/Pr = 0.1/0.1/1 mW. **c** Sketch of three Gaussian laser spots on the sample with the wide blue spot representing the probe (1/*e*^2^ = 150 μm, FWHM of 88.3μm), the central red spot is the pump-1 (1/*e*^2^ = 17 μm, FWHM of 10 μm), and the red spot on the right is the pump-2 (1/*e*^2^ = 9 μm, FWHM of 5.3 μm) that can be shifted horizontally. The top sketch shows a frontal view of the sample, and the bottom sketch shows the normalized intensity profile simulation of the involved beams of 38 *μ*m separation. **d** Experimental time traces of auto-oscillations for the case of the pump-2 spot shifted horizontally by 50 μm away from the pump-1 position. **e** First harmonic range of the fast Fourier transform spectrum of the traces presented in (**d**), measured for 10 min. Each separate pump beam induces auto-oscillations with different frequencies. When both pumps are applied together (green, bottom), two frequencies are observed at positions close to those of the single-pump beam cases, indicating no averaging and thus no synchronization. **f**, The measured synchronization range presented for a pump-2 shift from  − 50 μm to  + 50 μm relative to pump-1 position. The synchronization radius is about 38 ± 3 μm measured between the pump-spot centers. The precision of the rotation screw determines the error bars.

To investigate the processes responsible for the observed synchronization between different auto-oscillators, we modified our experimental setup by adding a second pump laser with the same photon energy as the first one. Figure 3c demonstrates the setting. We use two tightly focused pump beams (shown by the two small red circles) on the semiconductor surface to excite separate auto-oscillations, and a wide, defocused probe beam (blue circle) to read out the average electron spin polarization dynamics. The spatial position of one of the pump beams, marked as pump-2, can be precisely shifted on the sample, while pump-1 is always fixed at the center of the probe.

The blue time trace in Fig. 3a demonstrates several minutes of the auto-oscillations at the pump-1 position (pump-2 is blocked), where non-decaying oscillations are observed. The corresponding FFT for a ten-minute time trace is then represented in Fig. 3b by the blue curve, showing the first harmonic peak at *f*_1_ = 91 mHz. The red colored time trace in the middle of Fig. 3a demonstrates a pump-2-only experiment, where pump-2 is, as an example, shifted by  − 25 μm away from the pump-1 position, and pump-1 is blocked. The signal intensity is shown on the same scale. It is smaller than that of the top trace of pump-1, which, in addition to the variation of the parameters of the sample, is also related to a weaker probe intensity at this spatial position, see the intensity profile variation at the bottom part of Fig. 3c. Figure 3b shows the corresponding FFT with *f*_2_ = 69 mHz, 22 mHz lower than at the pump-1 position, by the red-colored spectrum. Finally, the green-colored trace at the bottom of Fig. 3a reflects the case when both pump-1 and pump-2 are open simultaneously. As shown in Fig. 3b, the corresponding FFT exhibits a single peak at *f*_sync_ = 80 mHz, located between *f*_1_ and *f*_2_, demonstrating clear synchronization. In that case, the FFT does not necessarily have to show the average frequency, but there is clearly only one peak in the 1st-harmonic range between *f*_1_ and *f*_2_. We emphasize that the green-colored time trace in Fig. 3a represents a novel type of oscillation that cannot be composed of a combination of two contributing signals. See the Supplementary section 3 for a wider frequency range than in Fig. 3a.

### Range of synchronization

We now continue shifting the pump beams apart. Figure 3d illustrates the case where the pump-1-only signal is presented again, but the pump-2-only signal is shifted 50 μm away from the pump-1 position (see the red-colored time trace). The green-colored bottom trace shows the signal with both pumps applied together. Figure 3e shows the corresponding FFT spectra with a main difference relative to Fig. 3b: the both-pumps-on case demonstrates two frequencies close to those of the positions of the contributing signals, indicating the absence of synchronization. In that example, one can argue that the green-colored trace in Fig. 3d represents the superposition of both measured signals. Conversely, this serves as an experimental confirmation that the probe detects the contributions of the two oscillators without synchronizing them.

Finally, Fig. 3f summarizes all measurements with the pump-2 position scanned across the range of −50 to +50 μm of relative shift between the pump spots. Here, the synchronization is present (1) if one observes a single frequency for the case of both pumps being applied together, and the synchronization is absent (0) if one detects both contributing single-pump frequencies under illumination with two pumps. We conclude that the synchronization range is 38 ± 3 μm taken between the central positions of the pump spots, or taking the spot sizes of the pump beams into account (given in the caption of Fig. 3), the beam separation is 25 ± 3 μm defined as the distance between the points where the spot intensities fall to 1/*e*^2^ of their maximum value. In Supplementary section 3, we additionally demonstrate the appearance of intermodulation products in the superposition signal without synchronization and the FFT spectra for all pump-to-pump distances shown in Fig. 3f.

## Origin of synchronization

For two spatially separated, auto-oscillating ENSSs, we only need a weak interaction that makes the instantaneous frequency (or phase) of one depend on the other. Let’s consider the most likely mechanisms present in an *n*-doped GaAs semiconductor that could provide a connection between remote places:

- Interaction by cross-illumination: it involves weak optical cross-talk between spatially separated regions, whereby pump light scattered from one excitation spot reaches the other and provides a weak common drive. In experiments employing a single pump spot (Fig. 2), this regime cannot be distinguished from other mechanisms, as different ENSSs are excited under nominally identical conditions. However, measurements performed with two independently controlled pump spots (Fig. 3) demonstrate a clear separation between the excitation regions, thereby excluding this cross-illumination mechanism as the origin of the observed effects. See Supplementary section 4 for experiments and further discussion on this mechanism.
- Nuclear spin diffusion: slow flip-flop-driven transport of nuclear polarization spreads Overhauser fields between the spots. The diffusion constant for GaAs is tiny (*D*_N_ ~ 10^−13^ cm^2^/s)^32^ and is in the same range as the one determined for our sample^30^, so the coupling is slow (seconds-minutes) but long-lived. Using the given *D*_N_ and the longest measured value of the nuclear spin relaxation time for our sample *τ*_N_ = 210 s^30^, leads to a diffusion length of ${L}_{{{\rm{N}}}}=\sqrt{{D}_{{{\rm{N}}}}{\tau }_{{{\rm{N}}}}}\approx 46$ nm.
- Electron-mediated coupling: hopping electrons can correlate nuclear baths in separated regions, altering nuclear spin polarization and effectively coupling the ENSS phases. For a given concentration of about 10^16^ cm^−3^ the diffusion coefficient is estimated to be about *D*_hop_ ≈ 1 cm^2^/s^33^, which taken with the spin relaxation time of *τ*_*s*_ = 120 ns leads to the length of *L*_hop_ ≈ 3.5 μm.
- Electron spin diffusion: spin-polarized free carriers created in one spot diffuse into the other, affecting the donor-bound electrons by the spin-exchange coupling^34^; the resulting Overhauser field feedback pulls the precession frequency and can lock the two oscillators. The spin-diffusion length in this case is given by ${L}_{s}=\sqrt{{D}_{{{\rm{s}}}}{\tau }_{{{\rm{s}}}}}$, where *D*_*s*_ is the spin diffusion constant and *τ*_s_ is the electron spin relaxation time. Taking into account the known values of *D*_s_ = 24 cm^2^/s ^35^ and *τ*_s_ = 120 ns measured for our sample^30^, one arrives at the diffusion length *L*_s_ ≈ 17 μm.

To provide additional support for the synchronization mechanism, we have additionally studied a similar semiconductor structure in which the electron concentration is reduced by about two orders of magnitude, from *n*_*e*_ = 39 × 10^15^ cm^−3^ to *n*_*e*_ = 0.5 × 10^15^ cm^−3^. Auto-oscillations could also be detected in this sample. However, the synchronization range is significantly reduced, with synchronization occurring only when the two pump spots overlap. This behavior is consistent with the shorter spin relaxation time in the low-density sample, *τ*_*s*_ ≈ 30 ns^36^, which leads to a reduced spin-diffusion length. Using *D*_*s*_ = 24 cm^2^/s, we estimate *L*_*s*_ ≈ 8.5 μm. See Supplementary section 5 for supporting experiments.

To summarize, based on the experimentally observed synchronization range of  ~  40 *μ*m and further supporting studies, we can suggest that the primary mechanism responsible for the synchronization is the spin diffusion of free electrons^33,34^.

To simulate it, we extend the model presented in the Refs. ^28,31,37^. The Bloch equation describing the spin polarization (**S**) precession in the external field (*B*_ext_) and the Overhauser field (*B*_N_) is extended by a term accounting for spin diffusion^38^:

1 $${{\bf{S}}}={{{\bf{S}}}}_{0}(x)+\frac{g{\mu }_{{{\rm{B}}}}{T}_{{{\rm{s}}}}}{\hslash }({{{\bf{B}}}}_{{{\rm{ext}}}}+{{{\bf{B}}}}_{{{\rm{N}}}})\times {{\bf{S}}}+{D}_{{{\rm{s}}}}{T}_{{{\rm{s}}}}\frac{{\partial }^{2}{{\bf{S}}}}{\partial {x}^{2}}.$$

Here, *D*_s_ is the spin diffusion coefficient, **S**_0_(*x*) is the average electron spin polarization in the absence of the magnetic field, created by the continuous wave pump with Gaussian or flat-top spatial profile, *μ*_B_ is the Bohr magneton, and *g* is the electron *g*-factor. *ℏ*/*μ*_B_*gT*_s_ is the half-width at half-maximum of the electron Hanle curve that is not influenced by dynamic nuclear polarization. *T*_s_ is the electron spin relaxation time, *ℏ* is the reduced Planck constant. For clarity, we restrict the diffusion to a single spatial direction (*x*).

The precession of the electron spin polarization about the total magnetic field changes the Overhauser field in time according to^37,39^:

2 $$\frac{d{{{\bf{B}}}}_{{{\rm{N}}}}}{dt}=-\frac{1}{{T}_{{{\rm{N}}}}}\left({{{\bf{B}}}}_{{{\rm{N}}}}-\hat{a}{{\bf{S}}}\right),$$

where $\hat{a}$ is the second-rank tensor describing the process of dynamic nuclear polarization. In In_0.03_Ga_0.97_As, the substitution of Ga atoms by a small fraction (3%) of indium induces significant and spatially non-uniform crystal deformations, extending beyond nearest neighbors and resulting in a highly inhomogeneous strain field due to its radially decaying character. This local strain leads to quadrupolar splitting of nuclear spin levels for all nuclear states with an absolute value of spin projection  > 1/2, causing the nuclear spins to align along local principal axes **h**_*i*_ of the electric field gradient tensor rather than along the external magnetic field. As a result, their contribution to the Overhauser field is given by **B**_Q_ = ∑_*i*_*a*_*i*_(**S** ⋅ **h**_*i*_)**h**_*i*_, where the sum runs over nuclei within the electron localization volume and *a*_*i*_ is proportional to the hyperfine coupling constant and the electron probability density at the *i*-th nucleus^40,41^. For an effectively isotropic distribution of these axes, this reduces to **B**_Q_ = *a*_N_**S**, allowing the hyperfine coupling tensor to be written in the simplified form $\hat{a}{{\bf{S}}}={b}_{{{\rm{N}}}}({{\bf{S}}}\cdot {{{\bf{B}}}}_{{{\rm{ext}}}}){{{\bf{B}}}}_{{{\rm{ext}}}}/| {{{\bf{B}}}}_{{{\rm{ext}}}}{| }^{2}+{a}_{{{\rm{N}}}}{{\bf{S}}}$^31^, which combines the contributions from quadrupole-unperturbed and quadrupole-perturbed nuclei, respectively. If a strongly anisotropic quadrupolar interaction dominated, one would expect suppression of auto-oscillations, which is not observed in our experiments^42^. The tensor has been simplified in the lowest-order approximation (for details, see Methods section, Ref. ^28^, and the corresponding Supplementary section 6).

**Fig. 4:**
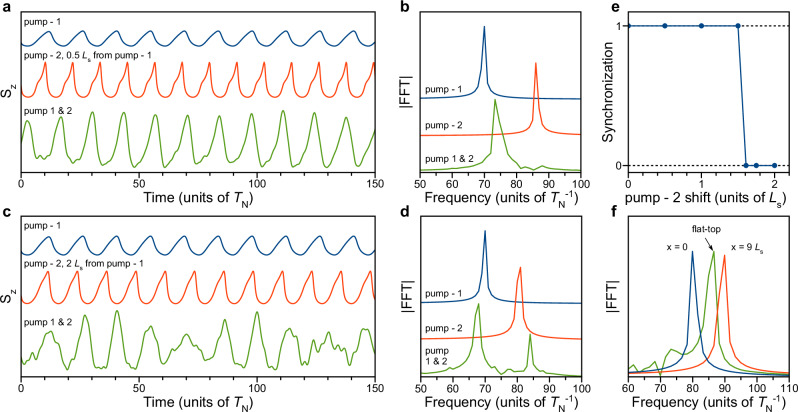
Simulation of synchronization. **a** Simulated time traces of auto-oscillations for three cases. The top (blue) trace corresponds to pump-1 only, the middle (red) trace to pump-2 only, shifted by 0.5*L*_s_ from the pump-1 position. The spatial width (FWHM) of both pump-1 and pump-2 is 0.25*L*_s_. The bottom (green) trace shows the combined excitation by pump-1 and pump-2. See Fig. 3c for pump labeling. Detection is performed with a spatially wide probe covering the range 0 − 10*L*_s_. **b** First harmonic of the fast Fourier transform spectra of the traces in (**a**). **c** Simulated time traces of auto-oscillations for three cases: the top (blue) trace corresponds to pump-1 only, the middle (red) trace to pump-2 only, shifted by 2*L*_s_ from the pump-1 position, and the bottom (green) trace to their combined excitation. **d** First harmonic of the FFT spectra of the traces in (**c**). **e** Extracted synchronization range for pump-2 shifts between 0 and 2*L*_s_ relative to pump-1. **f**, Synchronization in simulation under flat-top pump excitation. The blue curve shows the first harmonic in the FFT for the spatially narrow pump-1 and narrow probe, the red curve corresponds to pump-2 and probe shifted by 9*L*_s_, and the green curve shows the resulting signal under flat-top pump excitation spanning 0 − 10*L*_s_. The detection is performed with a spatially narrow probe at 9*L*_s_.

To account for sample variation, we introduce a spatial distribution of the hyperfine interaction parameters (see Supplementary section 6). When the system is excited by a spatially narrow Gaussian pump, auto-oscillations emerge, as shown in blue in Fig. 4a, with their frequency extracted via FFT (blue curve in Fig. 4b). Excitation by pump-2, positioned 0.5*L*_s_ away from pump-1, produces a modified oscillation pattern due to the inhomogeneous hyperfine coupling, with the corresponding frequency shift illustrated in red in Fig. 4b (*L*_s_ is the spin diffusion length.) When both pump-1 and pump-2 are applied simultaneously, the auto-oscillations occur at an intermediate frequency between *f*_1_ and *f*_2_, as seen in Fig. 4b (green). This behavior represents a clear manifestation of synchronization of auto-oscillations mediated by spin diffusion.

Synchronization mediated by spin diffusion is sensitive to the spatial separation between pump-1 and pump-2. To illustrate this, pump-2 was placed 2*L*_s_ away from the position of pump-1. The auto-oscillations induced by pump-2 are shown in red in Fig. 4c, with their corresponding frequency indicated in red in Fig. 4d. When both pumps are applied simultaneously, the FFT exhibits two distinct peaks close to *f*_1_ and *f*_2_, as shown in Fig. 4d, with the corresponding time trace also presented in Fig. 4c. The emergence of two distinct frequencies under simultaneous excitation by pump-1 and pump-2 is a clear indication of the loss of synchronization. Figure 4e shows the synchronization range versus pump-2 shift. Synchronization persists up to 1.5*L*_s_, consistent with experiment (Fig. 3f). The simulations reveal that the difference between the maximum and minimum values of the hyperfine coupling parameter significantly affects the synchronization threshold (see Supplementary section 6). So, for more similar initial frequencies, the synchronization range expands.

For completeness, we also simulated synchronization under a flat-top pump covering the spatial range from *x* = 0 to *x* = 10*L*_s_. To illustrate the spatial variation of the hyperfine interaction, we show the auto-oscillation frequencies *f*_1_ and *f*_2_ obtained for a spatially narrow pump placed at *x* = 0 and *x* = 9*L*_s_, respectively (blue and red curves in Fig. 4f). Under flat-top excitation, the auto-oscillation frequency locks to an intermediate value between *f*_1_ and *f*_2_ across the entire range 0≤*x*≤10*L*_s_ (see Fig. 4f)). Flat-top excitation demonstrates that spin diffusion promotes global synchronization across an inhomogeneous sample, consistent with the experimental observations.

Together with the agreement between experiment and simulation, the simulation results establish electron spin diffusion as the microscopic mechanism underlying the collective synchronization of auto-oscillations. Each donor electron is localized within a Bohr radius of about 11 nm and is coupled to approximately 10^6^ nuclear spins forming a single auto-oscillator. Free electrons passing through the region interact with donor-bound electrons via spin exchange, thereby transferring spin polarization. The spin polarization carried by free electrons diffuses over a much larger characteristic length scale (about 38 μm), effectively coupling and synchronizing multiple donor-based oscillators without requiring donor-to-donor electron hopping, nuclear spin diffusion, and interaction by cross-illumination. Within the electron spin diffusion mechanism, different regimes of frequency adjustment between coupled oscillators are possible, including 1:1 frequency locking, frequency pulling, and the free-running regime, which are shown in Supplementary Fig. S3.

Finally, to obtain a more complete picture, we also determined the diffusion length using the independent method of time-resolved magneto-optical Kerr rotation microscopy (see the Method section and the Supplementary section 7). The obtained spin diffusion value of *D*_s_ = 28 ± 1 cm^2^/s is very close to the previously reported one *D*_s_ = 24 cm^2^/s ^35^ cited above. This corresponds to a diffusion length of *L*_s_ = 18.3 ± 0.3 μm in our sample, confirming the observations.

## Discussion

Building on our previous report of the robust auto-oscillations in electron-nuclear spin systems in InGaAs^28^, our results establish that spatially separated electron-nuclear spin systems in a semiconductor can mutually synchronize through electron spin diffusion, locking their oscillations over mesoscopic distances. This collective phase persists under wide-area excitation, in which an extended ensemble of auto-oscillators contributes to a single synchronized state despite environmental variation. Although auto-oscillations may arise even in a single localized electron-nuclear spin subsystem, we show here that spin diffusion synchronizes a macroscopic ensemble of such auto-oscillators.

Importantly, the emergence of such macroscopic synchronization is itself the key factor that stabilizes the auto-oscillations, protecting them against fluctuations and disorder that would otherwise disrupt phase-stable dynamics. Demonstrating that diffusive spin currents can mediate both correlations over mesoscopic distances and enhanced stability highlights a new route for engineering robust networks of coupled spin oscillators in solid-state platforms. Furthermore, integrating electrostatic gating or lateral drift control would convert this passive non-local link into an actively tunable coupling channel. Such controllable networks may serve as a basis for analog neuromorphic architectures, in which information is processed via collective phase dynamics rather than discrete logic states. Beyond deepening our understanding of non-equilibrium collective dynamics, spin-mediated synchronization offers prospects for spintronic architectures and on-chip time-crystal-based devices.

## Methods

### Sample

The main sample under study is a 10 *μ*m thick InGaAs epilayer with 3% indium content. It was grown by molecular-beam epitaxy on a GaAs substrate and is homogeneously doped by Si atoms. A majority of the GaAs substrate is etched away. At low temperatures (*T* ≈ 2 K), the electron concentration is at *n*_*e*_ = 39 × 10^15^ cm^−3^, as thoroughly determined in ref. ^30^. An important effect of the indium incorporation is the strongly enhanced quadrupole splitting, leading to local nuclear fields of *B*_*Q*_ = 20 mT^30^. This value is an order of magnitude larger than in low-stressed GaAs structures^41^ and two orders of magnitude larger than in GaAs epilayers with a quadrupole-free local field, given purely by the dipole-dipole interaction of the nuclei^43^. The second sample used in this work is the 4 μm thick InGaAs epilayer with 3% indium content and an electron concentration of *n*_*e*_ = 0.5 × 10^15^ cm^−3^^36^. The sample with the low electron density is only used for the test of synchronization presented in Supplementary section 5. All other results are presented for a sample with the high electron density. Additional details are provided in the Supplementary section 1.

### Setup

We use a continuous-wave laser diode emitting at 1.579 eV (785 nm) as the pump laser, which is then routed through a quarter-wave plate to create circular polarization. The linearly polarized probe laser is produced by the continuous wave Ti: Sapphire ring-laser and is fixed at the 1.426 eV (869.4 nm). Both lasers are combined on a non-polarizing beam splitter and are focused by a single lens at the sample. Depending on the experiment, we also split off a portion of the pump laser to produce an additional pump beam that hits the sample at an angle of approximately 30 degrees. For the two-pump version presented in Fig. 3, we use an additional beam as a pump-1, which is fixed at the center of the probe beam at the sample. The second part of the pump beam, which is routed through the rotatable NPBS and combined with the probe, is then referred to as a pump-2. The NPBS controls the pump-2 position at the sample by the tip-tilt-rotation stage (TTR001 Thorlabs). For the experiment, presented in Fig. 1, we use only one pump beam and shift the whole cryostat by the micrometer stages. For the experiment in Fig. 2, the pump-1 beam is going through the *π*Shaper (AdlOptica Focal-*π*Shaper), which converts the Gaussian beam to a flat-top profile. The position of that beam is fixed at the sample, while the probe position is shifted within the pump.

The sample is mounted in a helium-flow cryostat at *T* = 6 *K* at the center of two orthogonal pairs of electromagnetic coils, generating magnetic field components *B*_*x*_ = − 1 mT and *B*_*z*_ = 0.176 mT.

The GaAs substrate of the sample completely absorbs the pump laser, while the probe laser is transmitted and then analyzed by the polarization bridge, which consists of a half-wave plate and a Wollaston prism. A balanced photodiode is used to measure the Faraday rotation of the linearly polarized probe.

We have used the Beam-master scanning multiple-knife-edge device (Coherent) to characterize the beam spot size at the sample position. It allows for measuring beam spot diameters as small as 3 with 0.1 μm resolution and larger beams up to 3 mm with 1 μm resolution.

More details and schematics are provided in the Supplementary section 2.

### TR-MOKE

For the spatial mapping and extraction of the spin diffusion coefficient, we use the following experimental configuration. Initial laser pulses with a temporal width of  ~ 35 fs are derived from a 60-MHz mode-locked Ti-sapphire oscillator. They are split into pump and probe paths and tuned by grating-based pulse shapers, resulting in pulses with a bandwidth of  ~ 0.5 nm and a temporal resolution of  ~ 1 ps. Pump pulses are modulated between left and right circular polarizations by an electro-optic modulator. The linearly polarized probe pulses are collinear with the pump and focused on the sample surface through a microscope objective. The FWHM diameters of pump and probe pulses are 3 ± 0.1 μm and 1 ± 0.1 μm, respectively. Kerr rotation is measured using balanced photodiodes connected to a lock-in amplifier, referenced to the modulation frequency. The probe polarization is resolved using a half-wave plate and a Wollaston prism. A mechanical delay stage adjusts the delay time between the pump and probe. The spatial overlap of the pump with the fixed and centered probe is adjusted through a lateral translation of the input lens of the beam-expanding telescope in the pump path^44–46^. All measurements are performed with the pump photon energy set to *E*_pu_ = 1.459 eV and the peak power density of 4.7 MW/cm^2^. The probe photon energy is *E*_pr_ = 1.43 eV with peak power density of 1.2 MW/cm^2^.

### Details on simulation model

The quadrupole unperturbed nuclear spin sublevels create the Overhauser field ${{{\bf{B}}}}_{{{\rm{N}}}}^{0}$ as in pure, unstressed GaAs. ${{{\bf{B}}}}_{{{\rm{N}}}}^{0}$ is aligned along the external magnetic field and compensates for the Zeeman splitting of the electrons in the external magnetic field. Due to the strong deformation caused by the indium incorporation, the spin of the *i*-th nucleus is oriented along the main local axis **n**_*i*_ of the tensor describing the quadrupole interaction rather than along the external magnetic field. The contribution of these nuclei to the total Overhauser field is **B**_Q_ = ∑_*i*_*a*_*i*_(**Sn**_*i*_)**n**_*i*_, where the summation is carried out over all quadrupole perturbed nuclei within the electron localization volume around a donor. For an isotropic distribution of the axes, the field can be written as **B**_Q_ = *a*_N_**S**. Therefore, $\hat{a}$ can be reduced to the simplified form: $\hat{a}{{\bf{S}}}={{{\bf{B}}}}_{{{\rm{Q}}}}+{{{\bf{B}}}}_{{{\rm{N}}}}^{0}={a}_{{{\rm{N}}}}{{\bf{S}}}+{b}_{{{\rm{N}}}}({{\bf{S}}}{{\bf{h}}}){{\bf{h}}}$, where *b*_**N**_ is the parameter of the hyperfine interaction between the electrons and the nuclei, and **h** is the unit vector of the externally applied magnetic field. The tensor components are: ${\hat{\alpha }}_{\alpha \beta }={a}_{{{\rm{N}}}}{\delta }_{\alpha \beta }+{b}_{{{\rm{N}}}}{{\mbox{h}}}_{\alpha }{{\mbox{h}}}_{\beta }$, with *α*, *β* = *x*, *y*, *z* coordinates.

The solution is integrated over a time window of  ~ 3*T*_s_, since the nuclear spin system has the relaxation time of *T*_N_ ≫ *T*_s_ and therefore experiences a stationary electron-nuclear polarization. This approach avoids numerical difficulties associated with directly solving the stationary Bloch equation.

The simulation algorithm consisted of three steps. In the first step, the electron spin polarization and its diffusion were calculated in the presence of the external magnetic field only. The stationary Bloch equation given by Eq. (1) leads to a divergent solution. To eliminate this divergence, the time-dependent Bloch equation was solved, and the resulting steady-state value was obtained by averaging over a time interval of 3*T*_*s*_. This procedure is justified because the nuclear spin relaxation time is much longer than the electron spin lifetime. The resulting spatial distribution of electron polarization defined the spatial profile of the Overhauser field, which exhibited auto-oscillations according to Eq. (2). In the final step, the diffusion of the electron spin polarization was computed in the total magnetic field (Eq. (1)), given by the sum of the external field and the Overhauser field obtained in the previous step.

All calculations were performed numerically in MATLAB using standard built-in libraries. The Overhauser field was calculated using the ode23 solver, while spin diffusion was simulated with the pdepe solver on a 600 × 4000 grid (space × time), covering a spatial range of 11.5*L*_s_ and a temporal range of 1000*T*_N_ with Neumann boundary conditions. The resulting signal included a transient region corresponding to the stabilization of auto-oscillations, containing approximately 250 points in the time series. The Fourier spectra presented in the main text were calculated after removing these transient points.

## Supplementary information

Supplementary Information

Transparent Peer Review file

## Data Availability

Data publicly available in a repository: 10.6084/m9.figshare.30580124.
