# Supplementary material for: Non-local synchronization of continuous time crystals in a semiconductor
Source: Nat Commun. 2026 Jul 22;17:6971. doi: 10.1038/s41467-026-75714-1 (PMC13392083; doi:10.1038/s41467-026-75714-1)
Supplement: Supplementary file 1 — Supplementary Information [file 41467_2026_75714_MOESM1_ESM.pdf]

Supplemental Materials for
Non-local synchronization of continuous time crystals in a semiconductor

S1. SAMPLES CHARACTERIZATION

Figure S1 shows the normalized photoluminescence (PL) of the used samples. Compared with previous publications [S1, S2], we have used the same sample but etched away the majority of the GaAs substrate to assess its effect on the behavior of the auto-oscillations. It has caused a shift in the PL emission to a higher wavelength. The dashed red line indicates the PL's initial position, while the solid blue line shows its position after the etching process. The etching procedure had no observable effect on the doping and overall CTC stability and operation, except that the main frequency of the CTC (and corresponding higher harmonics) has been decreased, from ~ 160 mHz to ~ 90 mHz. The green trace represents the PL of the low-doped sample with the black arrow marking the corresponding low energy position of the probe corresponding to a wavelength of 861.5 nm.

S2. EXPERIMENTAL SETUPS

The experimental setup for a two-pump configuration, presented in Fig. 3 of the main text, is shown in Fig. S2a.

We use the continuous wave laser diode emitting at 1.579 eV (785 nm) for the pump laser, which is then routed through the laser power stabilizer (NEL03A Thorlabs). We split the pump beam into pump-1 and pump-2 using NPBS. For the pump-1 beam, we use a lens with $f = 35$ mm, a 20 μm pinhole, and collimate the beam with a 150 mm lens. After that, we have beam routing to $\lambda/2-$Glan-Taylor (GT)$-\lambda/4$ with wide apertures (15 mm) and focus it at the sample using an achromatic lens with a focal length of $f = 100$ mm. The beam is directed at an angle of approximately 30° and is focused to a diameter of 17 μm at a $1/e^2$ intensity roll-off. The FWHM is the diameter at $1/e^2$ times $\sqrt{\ln 2}/\sqrt{2}$.

The pump-2 is prepared by using 35 mm lens, then 20 μm pinhole, and 150 mm collimating lens. It is then going through GT$-\lambda/4$ with wide apertures (pupil diameter 15 mm) to the NPBS, where it is combined with the probe beam path.

The linearly polarized probe laser is created by the continuous wave Ti:Sapphire ring-laser and is fixed at the energy of 1.426 eV (869.4 nm) for a high electron density sample, and at the energy 1.439 eV (861.5 nm) for the low electron density sample.

The pump-2 and probe beams are focused by a single achromatic lens with a 60 mm focal length onto the sample.

The 10 μm (or the 4 μm one for the second sample) epilayer and the remaining GaAs substrate absorb the pump laser entirely. In contrast, the probe laser is transmitted and then analyzed by the polarization bridge, which consists of a half-wave plate and a Wollaston prism. A balanced photodiode is used to measure the Faraday rotation of the linearly polarized probe, as shown in Fig. S2a. The differential signal is digitized with 4 ms time steps and stored on the PC. All presented time series in the main text and Supplemental materials were smoothed using a 20-point moving average.

For the measurements of sample inhomogeneity (see Fig. 1), we block the pump-1 beam (keep the pump-2 beam on) and shift the cryostat using the μm stages.

For the flat-top pump experiment (see Fig. 2), we use the scheme shown in Fig. S2b. Here, the pump-2 beam is blocked, while the probe is routed in the side entrance of the NPBS to be shifted at the sample. Pump-1 is transformed using the πShaper to produce the flat-top beam at the sample.

To recalculate the shift of the pump by rotating the stage with NPBS in Fig. S2a, one has to consider the following: a shift at the sample $x = f \cdot 2 \cdot \tan(\theta)$, and for small θ, $\tan(\theta) \approx \theta$. f - is the focal length of the lens, in our case 60 mm. One full rotation of the μm-screw at the rotation stage corresponds to 500 μm scale reading and 1.5° of rotation about vertical axis, or 1 μm$=$ 3×10^{-3} deg. Therefore, a shift at the sample $x = f \cdot 3 \times 10^{-3} \cdot 2\pi/180 \cdot s[\mu\text{m}]$, with s - being the micrometer scale of the rotation screw. With $f = 60$ mm it is: $x = 2\pi \cdot s[\mu\text{m}]$. The scaling factor was further confirmed by placing the μm-scale ruler at the sample position and reading the spot shift on the camera.

As the probe beam also passes through the same (rotating) NPBS, it is expected to exhibit a weak horizontal shift with NPBS rotation, which is an order of magnitude smaller than that of the reflected pump beam and is therefore irrelevant in all experiments.

S3. EXTENDED DATA

Depending on the symmetry and strength of coupling, two auto-oscillators can produce the following response in the FFT:

- **1:1 synchronization:** a single, narrow line at f_{sync}; harmonics at integer multiples of f_{sync}; intermodulation sidebands largely vanish, see Fig. S3a. The synchronization frequency between two auto-

oscillators is determined by the balance of coupling strengths [S3], nonlinear frequency shifts, dissipation, and nonreciprocal feedback [S4].

$$f_{\mathrm{sync}} \approx f_1 + \frac{\kappa_1}{\kappa_1 + \kappa_2}(f_2 - f_1), \tag{S1}$$

where κ_i are effective coupling weights. Thus, the synchronized frequency interpolates between the two natural frequencies, leaning toward the one with stronger coupling, higher amplitude, or lower damping.

- **Frequency pulling:** the two peaks move toward each other and broaden. Partially seen in see Fig. S3b for the fundamental frequencies and higher harmonics.
- **Free-running:** two distinct peaks at f_1 and f_2; in a combined signal you may also see intermodulation products $(nf_1 \pm mf_2)$ and a time-domain beat at $(|f_1 - f_2|)$, see Fig. S3c, Fig. S4, and Fig. S5a.

Figure S3a shows the extended frequency range, demonstrating how the synchronization is seen for the first three harmonics of the signal. The green colored trace demonstrates peaks in the middle between the pump-1-only and pump-2-only harmonics.

Figure S3b shows the extended frequency range in the case where the synchronization is broken. The green-colored trace demonstrates a double-peaked structure for each harmonic, with one peak corresponding to the frequency of pump-1 and the second peak being close to the pump-2-only position (although slightly shifted).

Figure S3c is the expanded view of Fig. S3b for the case without synchronization with vertical lines marking the main harmonics, the beat, and intermodulation products.

Figure S4 demonstrates the FFT peaks positions for all cases of two-pump beam separations, presented in Fig. **??**f of the main text. The presence of a single peak in the both-pumps-on case indicates synchronization, although the peak position does not necessarily coincide with the exact average of the individual frequencies. In the absence of synchronization, two distinct peaks corresponding to the contributing frequencies, along with their intermodulations, can be observed.

S4. INTERACTION BY CROSS-ILLUMINATION

A pump light scattered from one spot could reach the other spot and provide a (very) weak common drive. This regime cannot be distinguished from the others in an experiment with a single pump, as shown in Fig. 2, where different ENSSs are excited using a single pump spot. Still, we can be certain of a clear separation between the spots in experiments with two pumps, as shown in Fig. 3, excluding this mechanism.

To demonstrate the influence of the cross-illumination, we have expanded both pump spots as shown in Fig. S5c. Figure S5a demonstrates exemplary FFT spectra at different pump-separations, while Fig. S5b summarizes the measurements. In that setting, the observed synchronization radius between the pump spot centers has increased to about $50 \pm 5\,\mu$m. Taking the spot sizes into account, this leads to a beam separation of $19 \pm 5\,\mu$m, defined as the distance between the points where the spot intensities fall to $1/e^2$ of their maximum values. This value is close to that observed for the spatially narrow pump spots of $25 \pm 3\,\mu$m, and gives additional support for our observations.

S5. SUPPORTING EXPERIMENTS

Temperature

It is crucial to establish the physical mechanism of synchronization. To further address this issue, we have performed measurements at two temperatures (6 K and 15 K), with the upper temperature being limited to about 15 K, as the auto-oscillations completely vanish at approximately 17 K [S1]. Interestingly, we found no significant difference in the synchronization range between 6 K and 15 K. This observation suggests that while the auto-oscillations themselves are highly sensitive to temperature, the underlying coupling mechanism remains relatively robust within this temperature window.

Probe influence on synchronization

As in all our experiments with two pumps, we use a wide probe to test the system, it is crucial to rule out the effect of the probe itself in the process of synchronization. First, the absence of probe influence is supported by the experiment shown in Figs. 3d,e. In this configuration, the two pump spots are separated by a distance exceeding the carrier diffusion length, so that the two regions generate independent auto-oscillations. When each pump is applied individually, two distinct oscillation frequencies are observed (Fig. 3e). When both pumps are applied simultaneously, both frequencies remain clearly visible in the Fourier spectrum. If the probe signal were merely averaging the response from the two regions, these two modes would collapse into a single effective frequency. The observation of two distinct spectral peaks therefore demonstrates that the probe detects the contributions of the two oscillators without synchronizing them.

To further support our claims, we have also performed an additional test: we placed the pump-separation at the edge of the synchronization range, with synchronization still present, and tested the synchronization behavior for different probe powers, see Fig. S6. The hypothesis we are testing is the following: if the probe power

provides an influence on the synchronization between pumps, reducing its power would affect the synchronization range. We observed that reducing the probe power by two orders of magnitude has no effect on synchronization, as evidenced by the FFT traces in Fig. S6c.

Electron concentration effect on synchronization.

To test the effect of electron concentration on the synchronization range, we have studied the emergence of auto-oscillations in a similar structure with a reduced electron concentration. For this, the electron density was reduced from $39 \times 10^{15}\,\mathrm{cm}^{-3}$ to $0.5 \times 10^{15}\,\mathrm{cm}^{-3}$.

The reduced electron concentration lowers the number of optically addressed donor-electron spin systems, reducing the number of participating oscillating components. To recover a sufficient signal-to-noise ratio, larger pump spots had to be used (pump-1 is $34\,\mu$m and pump-2 is $50\,\mu$m). This increases the number of locally excited and mutually synchronized components within each pumped region, but potentially increases the optical cross-illumination effects in the two-pump configuration. Therefore, the measured center-to-center synchronization edge in the low-density sample should be regarded as an upper bound on the intrinsic non-local spin-mediated range rather than as a direct diffusion length, see Fig. S7.

For the lower-electron-density sample, the synchronization boundary shifts to a center-to-center pump separation of about $35\,\mu$m, despite the substantially larger pump diameters of $34\,\mu$m and $50\,\mu$m. At this separation, the corresponding $1/e^2$ excitation contours still overlap by approximately $7\,\mu$m. Thus, in contrast to the high-density sample, where synchronization persists across a finite dark gap of about $20\,\mu$m between the $1/e^2$ contours, the low-density sample loses synchronization already in an overlapping-beam geometry. This demonstrates a strong reduction of the effective non-local coupling range and supports our conclusion that synchronization is mediated by electron spin diffusion.

S6. SPIN DIFFUSION CALCULATIONS

Figure S8a shows the spatial profile of the flat-top pump covering the region from $x = 0$ to $x = 10L_\mathrm{s}$ used for calculations of Fig. 4f in the main text. The corresponding components of the electron spin polarization in the external magnetic field $B_x = -1\,\mathrm{mT}$ tilted by $\alpha = 10°$ with respect to the sample plane, S_x (black), S_y (blue), and S_z (red), are shown in Fig. S8b.

For calculations of Figs. 4a-e, the excitation of two Gaussian pumps with a full width at half maximum of $0.25L_\mathrm{s}$, separated by a variable distance, is used. Figure S8c illustrates their spatial profile. The components of the electron spin polarization, S_x (black), S_y (blue), and S_z (red), are shown in Fig. S8d for excitation by the two Gaussian pumps in the external magnetic field $B_x = -1\,\mathrm{mT}$ tilted by $\alpha = 10°$ with respect to the sample plane.

Figure S9a shows the spatial dependence of a_N (red) and b_N (blue) under flat-top pump excitation (green), used for calculations of Fig. 4f in the main text. Figure S9b presents the corresponding spatial dependence of the first harmonic calculated for the hyperfine interaction parameters shown in panel **a** in the absence of spin diffusion in the external magnetic field $B_x = -1\,\mathrm{mT}$ tilted by $\alpha = 10°$ with respect to the sample plane. Figure S9c displays the spatial dependence of a_N (red) and b_N (blue) under excitation by two Gaussian pumps separated by a distance of $2L_\mathrm{s}$, with the green curve indicating the respective pump profiles. The relative values of the hyperfine interaction parameters were increased compared to those used in the calculations with the flat-top pump. This adjustment was necessary because the spin polarization generated by a spatially narrow Gaussian pump is smaller than the average polarization produced by the spatially broad flat-top pump. To ensure that the resulting frequency of the first harmonic in the Fourier spectrum remains comparable between the two calculation types, the hyperfine coupling constants were adjusted.

To highlight the dependence of the synchronization range on the hyperfine coupling span, we present an additional calculation. Figure S10 shows an example of the spatial distribution of hyperfine interaction parameters. The difference between the minimum and maximum values of a_N is now increased up to 50 mT, which is larger than in the distribution used for the calculations presented in the main text. In this case, the synchronization threshold shifts down to $0.65\,L_s$.

S7. SPATIAL KERR MICROSCOPY

Figures S11a and S11b demonstrate the spatial maps of the time-evolution of the excited spin polarization measured by the pump-probe scanning Kerr microscopy at time delays of 0.2 and 1.8 ns, exemplary. The images directly reveal the spatial dependence of spin diffusion after the excitation pulse of 1 ps duration.

Figure S11c demonstrates the exemplary space-resolved spin polarization, derived from the measured Kerr rotation at 0.2 ns time delay in the spatial Kerr microscopy. As shown, the space-resolved polarization consists of two Gaussian profiles: a narrow one is produced by the pump pulse at zero time delay and becomes broader with increasing time delay. In contrast, the wider profile is related to the signal coming from the previous-pump-pulse contributions, similar to Ref. [S5]. We have fitted both of them separately. The corresponding dependencies of the FWHM^2 versus time delay are shown in Figs. S11d and S11e, yielding similar values of diffusion coefficients: $D_s = 28 \pm 1\,\mathrm{cm}^2/\mathrm{s}$ and $D_s = 35 \pm 6\,\mathrm{cm}^2/\mathrm{s}$,

respectively.

Furthermore, following the discussion in Ref. [S5], one can estimate the influence of hot electrons. As excitation occurs at much higher energies than the detection, the pump excites hot electrons that relax/cool down with an accelerated diffusion constant. According to Ref. [S5], one expects the electrons to cool down in approximately 400 ps, while the diffusion constant is changing exponentially (decreasing) within that time. So, over the ~ 400 ps cooling window, the enhanced diffusion increases the excited spin-packet width by $\approx 3\,\mu$m. The hot-electron phase provides a slight additional contribution to diffusion, but in the grand scheme, the longer, cooler diffusion is the dominant length scale.

In the experiments presented in the main text, the excitation energy was chosen to be close to the probe energy, allowing us to measure the diffusion constant of the cooled electron spins.

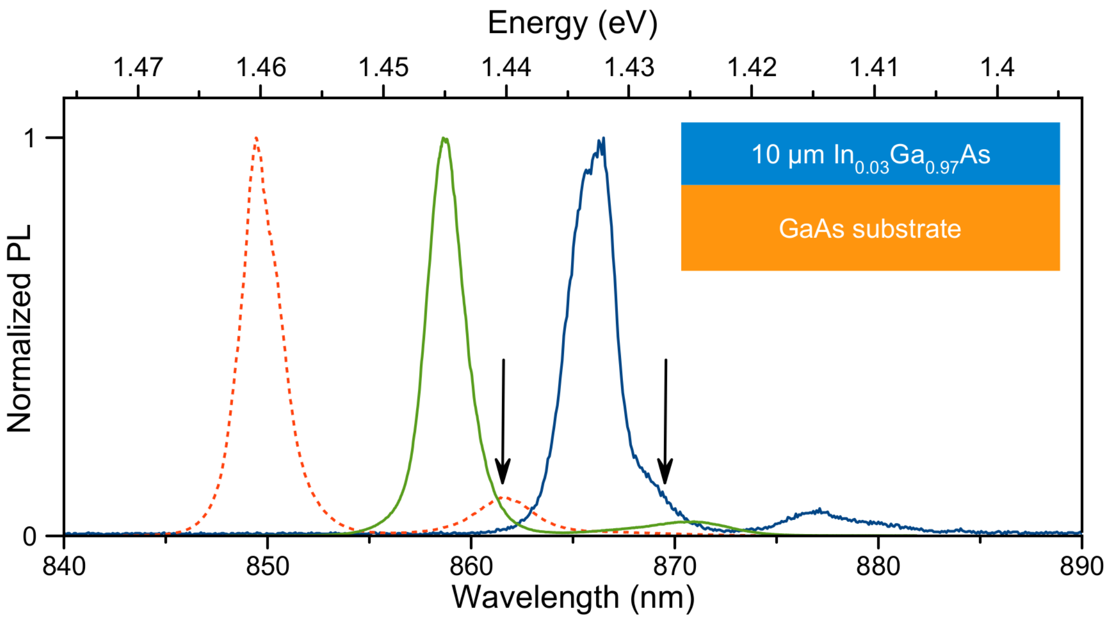

Supplementary Figure S1. **PL and Sample.** Normalized PL of three samples. The red dashed line shows the PL of the high-doped sample on the thick GaAs substrate. The blue solid line shows the sample with a reduced GaAs substrate thickness. The black arrow shows the position of the probe wavelength. Pump is at $E_{\mathrm{exc}} = 1.579\,\mathrm{eV}$ (785 nm), and the probe $E_{\mathrm{pr}} = 1.426\,\mathrm{eV}$ (869.4 nm). The inset shows the sample structure with a 10 μm epilayer and a GaAs substrate. The green trace shows the PL position of the low-doped 4 μm-thick epilayer used for additional studies. Black arrow shows the probe position at 861.5 nm.

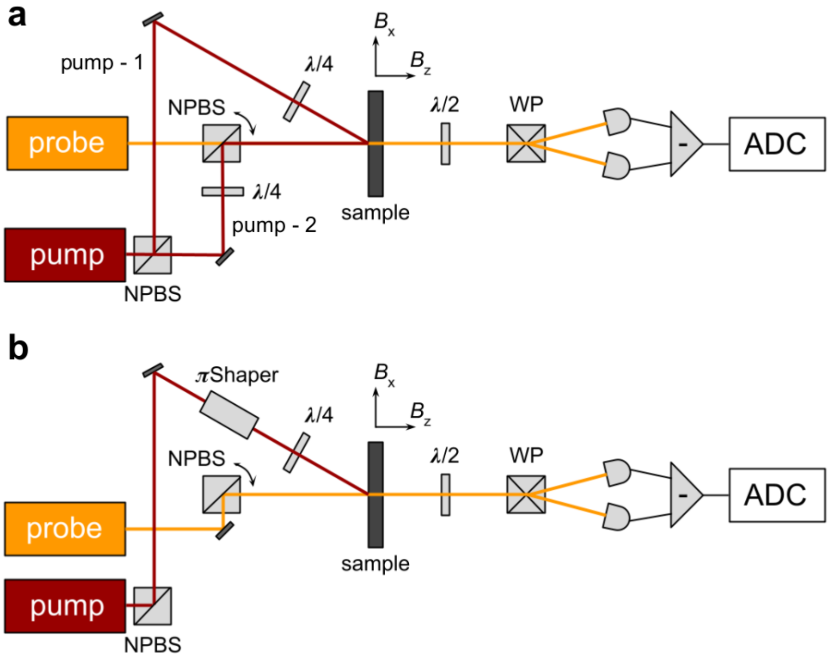

Supplementary Figure S2. **Schematics of the experimental setups. a**, The setup for the two-pump configuration. The additional pump beam (pump-1, coming under an angle) is blocked for the measurements presented in the main text Fig. 1, and the μm-stages move the cryostat. For the two-pump experiments presented in Fig. 3 of the main text, pump-1 is focused on the center of the probe beam while pump-2 can be shifted at the sample. **b**, Configuration for a flat-top pump measurements, presented in main text Fig. 2. The elements are: a non-polarizing beam splitter (NPBS), $\lambda/2$ and $\lambda/4$ are the half- and quarter-wave plates, a Wollaston prism (WP), and a πShaper to convert the Gaussian profile to a flat-top beam profile. The rotation of the NPBS in front of the sample was used to shift the spot reflected on the NPBS onto the sample. The energy of the pump laser is $E_{\rm pu} = 1.579\,\rm eV$ (785 nm), and of the probe $E_{\rm pr} = 1.426\,\rm eV$ (869.4 nm) or $E_{\rm pr} = 1.439\,\rm eV$ (861.5 nm), depending on the sample.

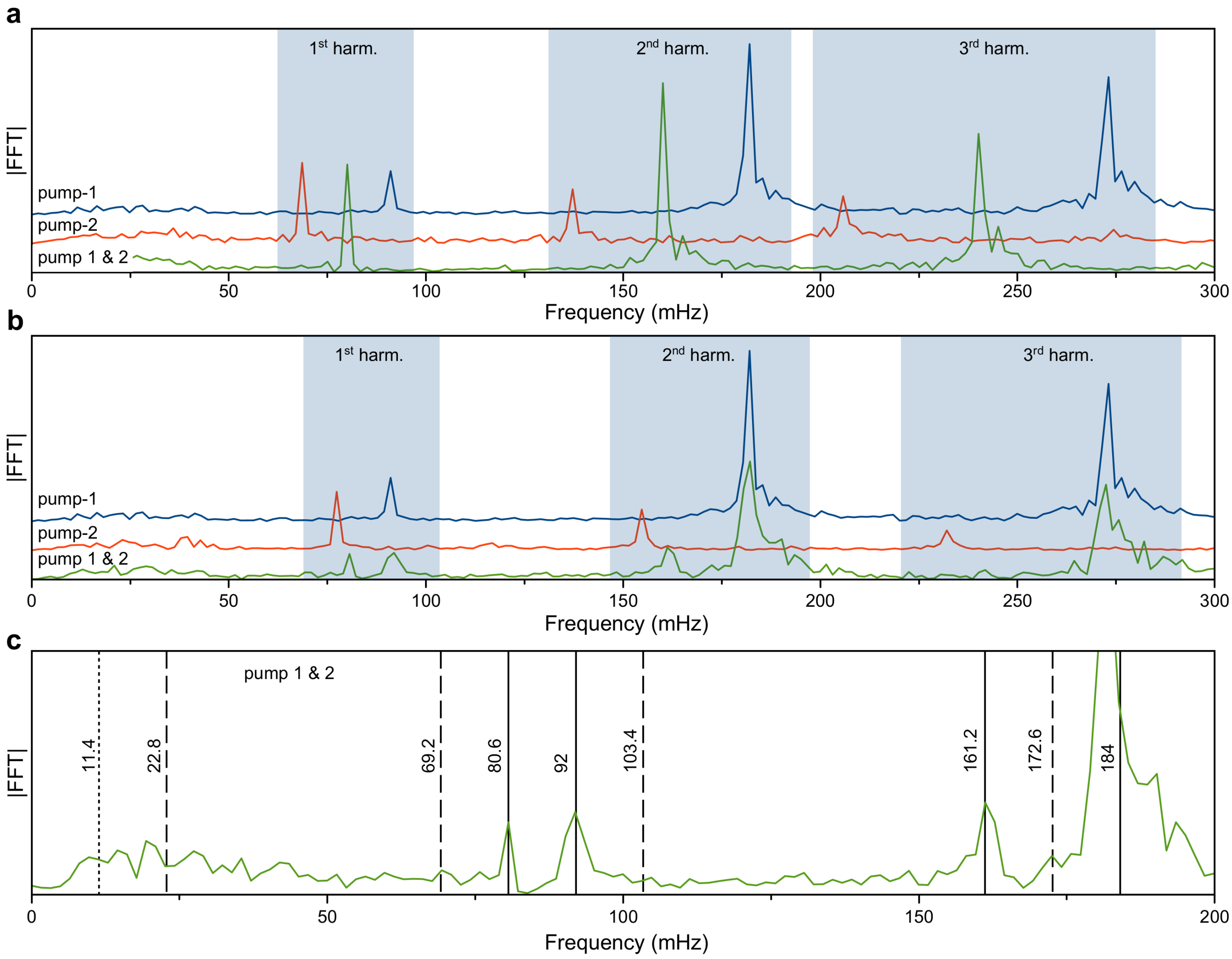

Supplementary Figure S3. **Extended FFT data. a**, Extended frequency range of the Fig. 3b demonstrating the range of the first three harmonics in the case of synchronization (1:1 synchronization). **b**, Extended frequency range of the Fig. 3e demonstrating the range of the first three harmonics in the absence of synchronization (free-running). **c**, Zoomed-in FFT for the case without synchronization, with the solid lines marking the main frequencies ($f_1, f_2, 2f_1, 2f_2$), a short-dashed line marking the beat (difference), and long-dashed lines marking the intermodulation products with $n, m \leq 2$.

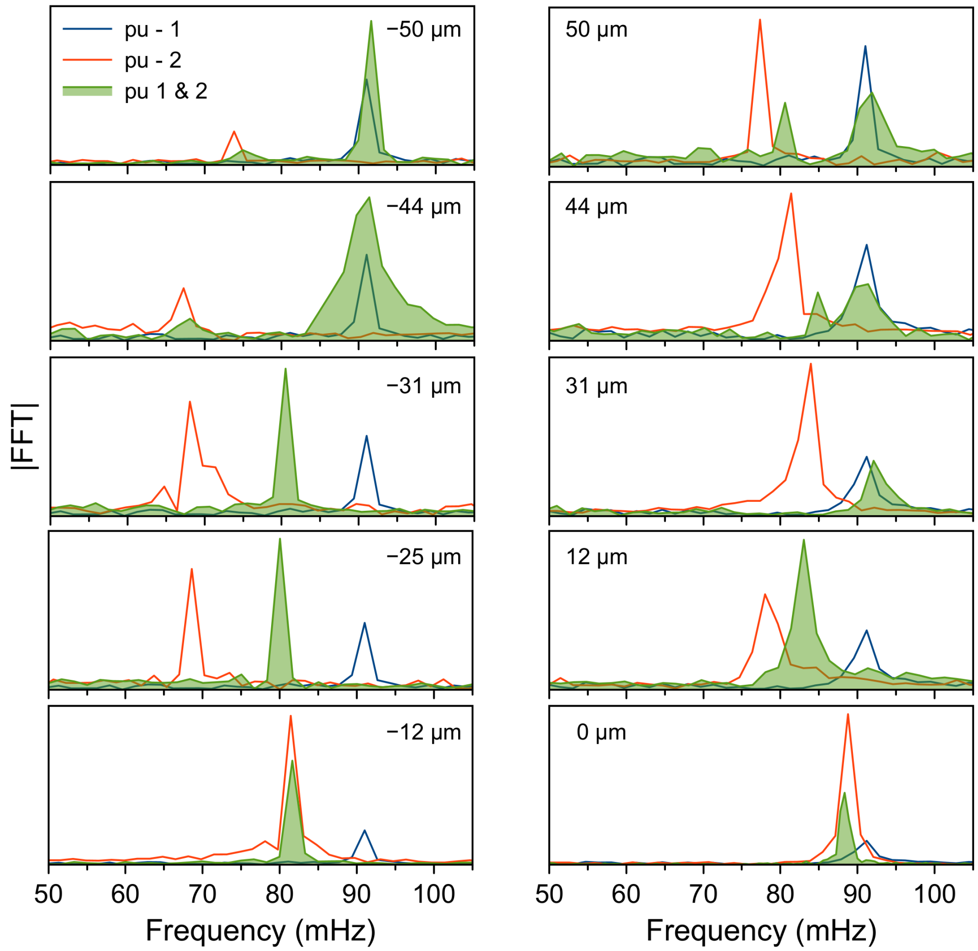

Supplementary Figure S4. **FFT at different pump separations.** FFT spectra for pump-1-only (blue), pump-2-only (red), and both pumps together (green shaded) cases for different pump-spot separations.

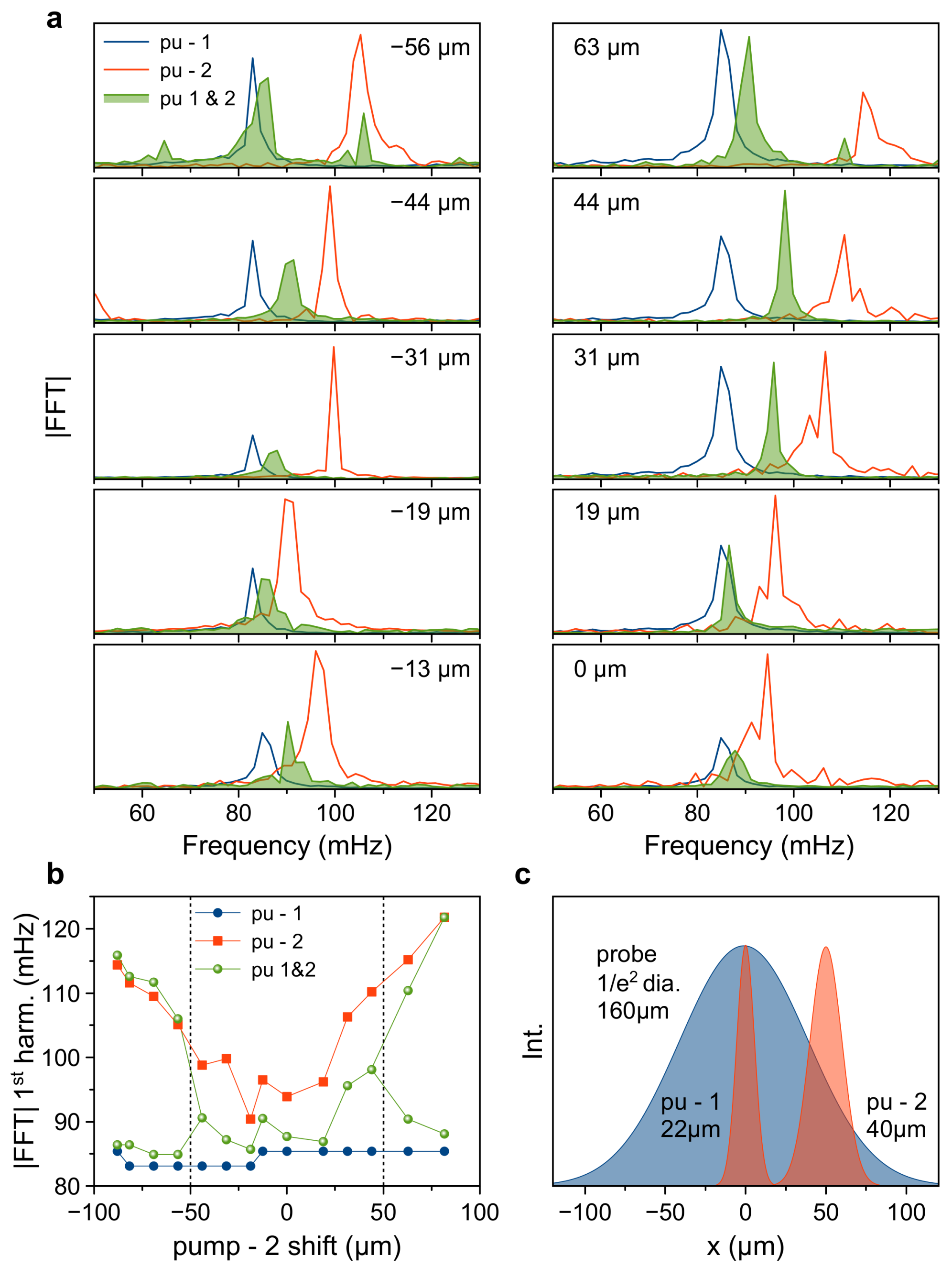

Supplementary Figure S5. **Synchronization with spatially wide beams.** **a**, FFT spectra for pump-1-only (blue), pump-2-only (red), and both pumps together (green shaded) cases for different pump-spot separations. **b**, A summarizing plot with points representing the central frequency of the first FFT harmonic. Vertical dashed lines at $\pm 50\,\mu$m indicate the approximate synchronization border. **c**, Simulation of the measured spot sizes with the probe beam diameter $160\,\mu$m at $1/e^2$ intensity roll off, blue shaded region. Pump-1 is placed at the center of the probe and has a diameter of $22\,\mu$m, while pump-2 is shifted by $50\,\mu$m away from pump-1 and has a diameter of $40\,\mu$m.

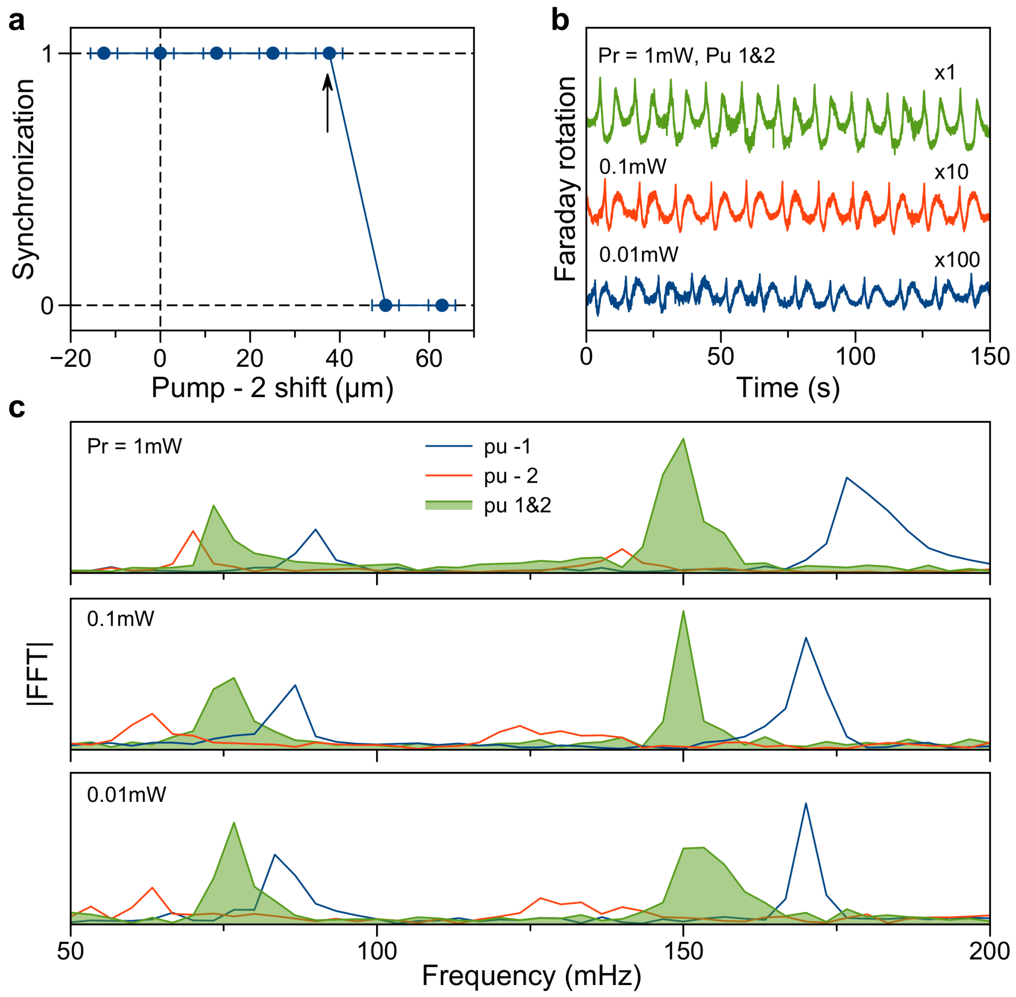

Supplementary Figure S6. **Probe power influence.** **a**, Synchronization range at probe power of 1 mW. The black arrow marks the position fixed for further tests. Error bars are determined by the micrometer screw's precision. **b**, Parts of time traces for three different probe powers (given in the panel) with the corresponding scaling factors measured with both pumps on (synchronization case). The lowest probe power of 0.01 mW is at the lower range of the sensitivity. **c**, FFT traces for pump-1-only (blue), pump-2-only (red), and both pumps on (green shaded) traces for three different probe powers. As seen, the peak positions are slightly shifted by reducing the probe power from 1 mW to 0.1 mW, but are not affected by further reductions. Overall, the synchronization is not affected as only one peak is observed for the both-pumps-on case.

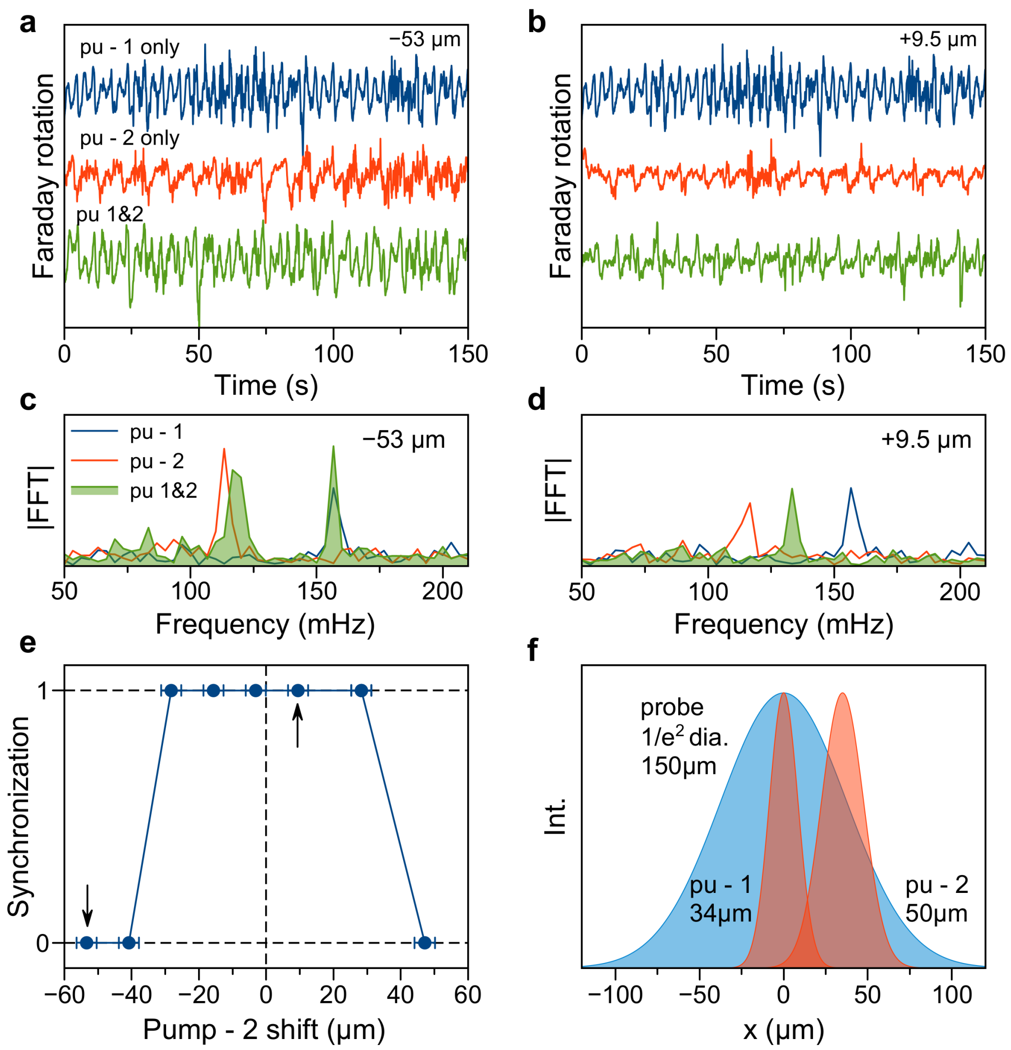

Supplementary Figure S7. **Reduced synchronization range.** Measured time traces for two different pump-separations: -53 μm (**a**) and +9.5 μm (**b**). Blue traces are for a pump-1-only excitation, red - for pump-2-only, and green for both pump on. **c** and **d** are the FFT spectra for the corresponding time traces of 10-minutes long accumulations. There is a clear difference in behaviour for the non-synchronized (**c**) and synchronized (**d**) case. **e**, the resulting synchronization range of about 35 μm. Error bars are determined by the micrometer screw's precision. **f**, Simulated Gaussian profiles of the contributing beams with the pump-beams separation of 35 μm.

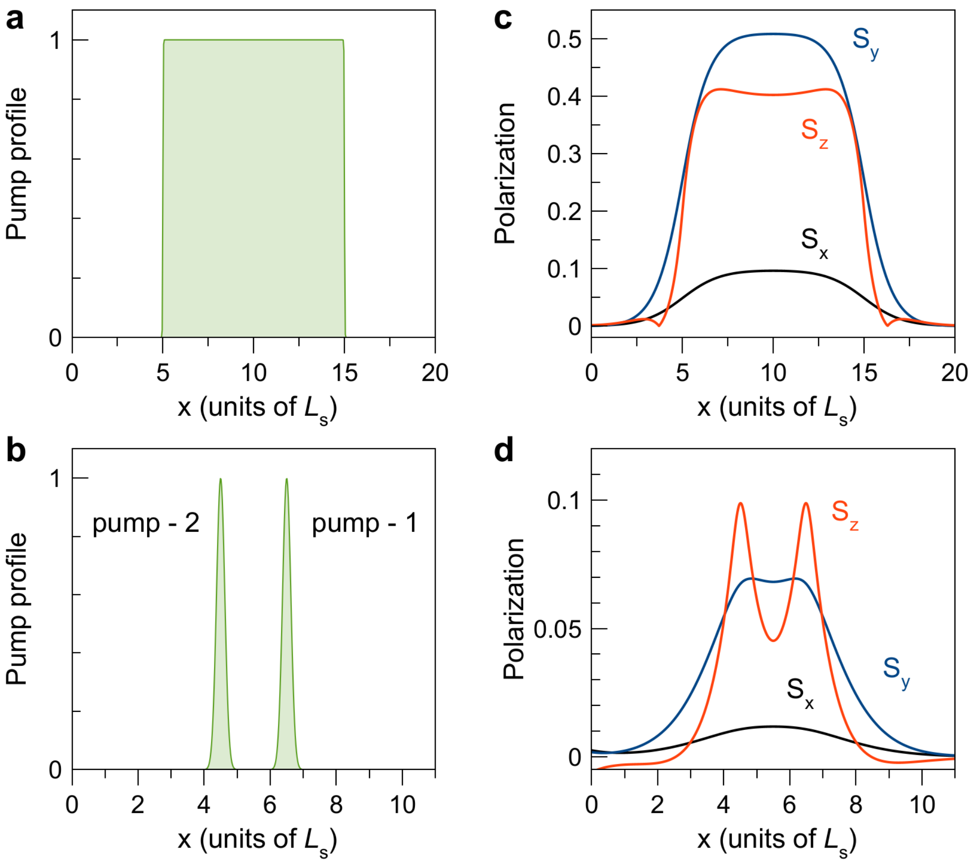

Supplementary Figure S8. **Electron spin diffusion in magnetic field.** Spatial profiles of the flat-top pump covering the range from $x = 0$ to $x = 10L_s$ (**a**) and two Gaussian pumps having FWHM of $0.25L_s$ (**b**) separated by a distance of $2L_s$. Electron spin polarization components: S_x (black), S_y (blue), and S_z (red), in a magnetic field of $B_x = -1\,\mathrm{mT}$ ($\alpha = 10°$) under excitation by the flat-top pump (**c**) and by two Gaussian pumps (**d**).

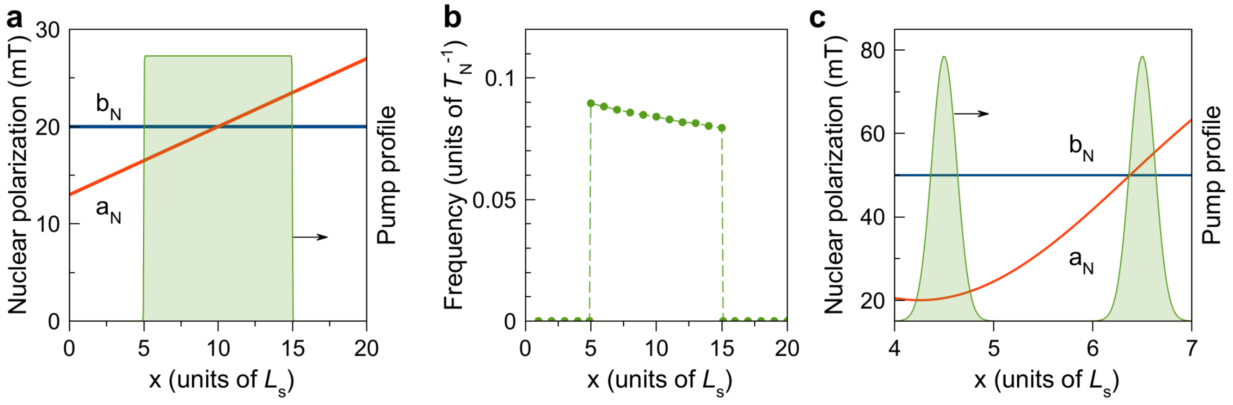

Supplementary Figure S9. **Spatial distribution of hyperfine interaction parameters.** **a**, Spatial dependence of a_N (red) and b_N (blue) under flat-top pump excitation; the green curve indicates the pump profile. **b**, Spatial dependence of the first harmonic for hyperfine interaction parameters shown in **a** without spin diffusion. **c**, Spatial dependence of a_N (red) and b_N (blue) under excitation by two Gaussian pumps separated by $2L_s$; the green curve indicates the pump profiles.

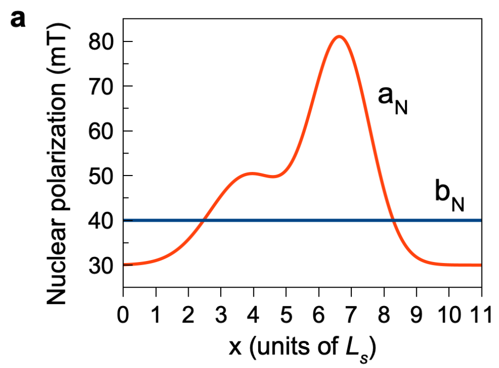

Supplementary Figure S10. **Spatial distribution of hyperfine interaction parameters.** **a**, Spatial dependence of a_{N} (red) and b_{N} (blue). **b**, Synchronization range for a_{N} and b_{N} from panel **a** for pump-2 shifts between 0 and $2L_{\mathrm{s}}$ relative to pump-1.

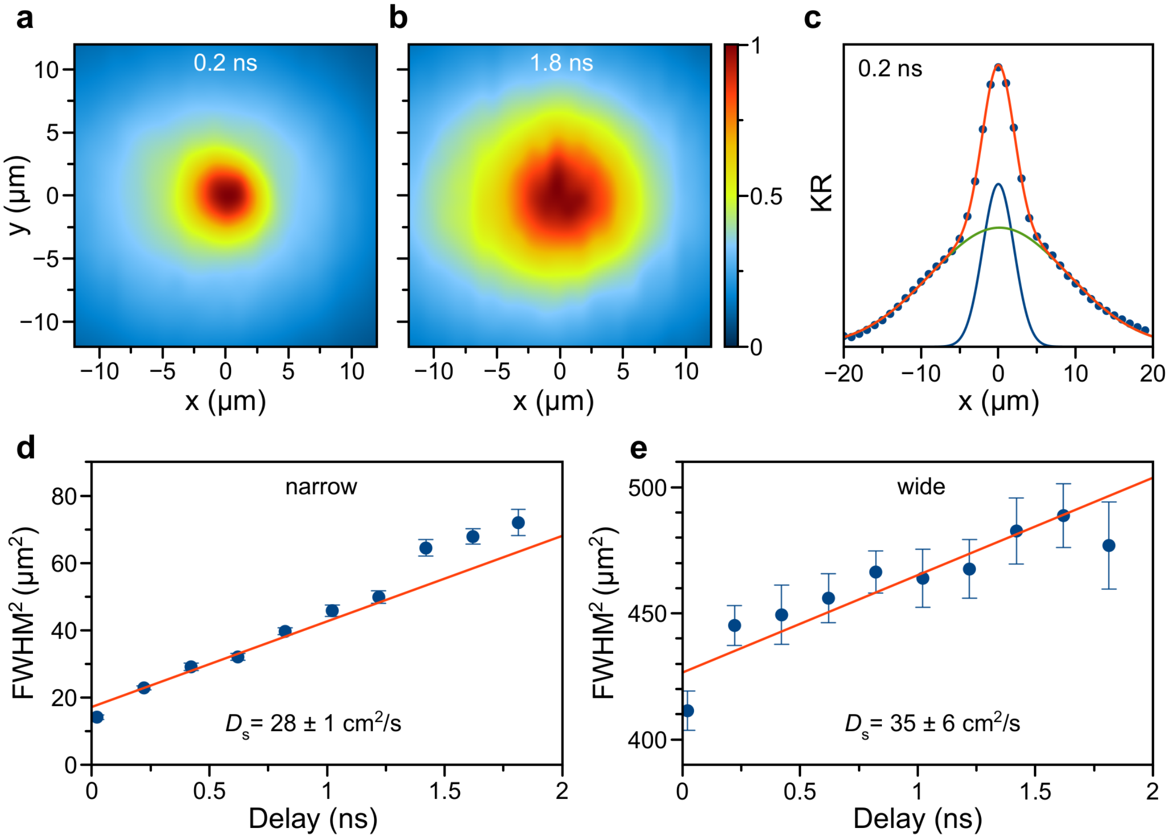

Supplementary Figure S11. **Full electron spin diffusion.** Contour plots for the time-resolved pump-probe Kerr rotation experiment with spatial mapping measured at time-delays of 0.2 ns (**a**) and 1.8 ns (**b**), demonstrating the evolution of the spatial profile of the excited spin polarization. The Kerr rotation amplitude is normalized, and its scale is shown by a color bar on the right. **c**, Exemplary double Gaussian fit of the spin polarization at the time delay of 0.2 ns. The blue data points are well-fitted by a narrow (blue line) and a wide (green line) Gaussian profile. The red line is the sum of both fits. **d**, Extracted time evolution of the narrow fit components in FWHM^2 versus time delay. The slope of the linear fit yields the spin diffusion coefficient $D_s = 28 \pm 1\,\mathrm{cm}^2/\mathrm{s}$. **e**, The same for the wide fit component, with $D_s = 35 \pm 6\,\mathrm{cm}^2/\mathrm{s}$.

SUPPLEMENTARY REFERENCES

[S1] A. Greilich, N. E. Kopteva, A. N. Kamenskii, P. S. Sokolov, V. L. Korenev, and M. Bayer. Robust continuous time crystal in an electron–nuclear spin system. *Nature Physics*, 20(4):631–636, Apr 2024.

[S2] Alex Greilich, Nataliia E. Kopteva, Vladimir L. Korenev, Philipp A. Haude, and Manfred Bayer. Exploring nonlinear dynamics in periodically driven time crystal from synchronization to chaotic motion. *Nature Communications*, 16:2936, 2025.

[S3] Y. Kuramoto. *Self-entrainment of a population of coupled non-linear oscillators.* Springer, Berlin, 1975.

[S4] Michel Fruchart, Ryo Hanai, Peter B. Littlewood, and Vincenzo Vitelli. Non-reciprocal phase transitions. *Nature*, 592(7854):363–369, Apr 2021.

[S5] T. Henn, T. Kiessling, W. Ossau, L. W. Molenkamp, D. Reuter, and A. D. Wieck. Picosecond real-space imaging of electron spin diffusion in GaAs. *Phys. Rev. B*, 88:195202, Nov 2013.
