# Supplementary material for: Non-local synchronization of continuous time crystals in a semiconductor
Source: Nat Commun. 2026 Jul 22;17:6971. doi: 10.1038/s41467-026-75714-1 (PMC13392083; doi:10.1038/s41467-026-75714-1)
Supplement: Supplementary file 2 — Transparent Peer Review file [file 41467_2026_75714_MOESM2_ESM.pdf]

nature portfolio

Peer Review File

Non-local synchronization of continuous time crystals in a semiconductor

Corresponding Author: Professor Alex Greilich

This file contains all reviewer reports in order by version, followed by all author rebuttals in order by version.

Version 0:

Reviewer comments:

Reviewer #1

(Remarks to the Author)
The paper reports on spatial correlation properties of self-oscillations in an optically pumped electron-nuclear spin system of a semiconductor sample. The work follows up from recent studies conducted by the same authors [Nature Phys 20 631, Nature Comm 16 2936], so there is some overlap. However, the subject of the present manuscript is quite distinct (focus on spatial correlations). The work reports nice experimental results and presents interesting physics phenomena. I broadly support publication upon adequate revision. My main general criticism is on the depth of experimental results and analysis. My feeling is that the study is somewhat superficial, reporting some experimental phenomena but without going deep enough in terms of understanding the underlying mechanisms. Some lack of details in understanding made sense for earlier reports on the subject [Nature Phys 20 631], but as research progress is made, one would expect to seem a more comprehensive study. In other words, it’s fine when the initial report discovering the self-oscillation effect leaves many details in the “yet to be understood” category, but one would expect more mature analysis for a third publication on the same experimental effect. There are also some claims, which I think are not properly supported by the data (e.g. phase spin coherence). The details are outlined below. Most of the comments below are linked to parts of the manuscript which are quoted as “”:

“based on the experimentally observed synchronization range of ∼ 40 μm, we can suggest that the primary mechanism responsible for the synchronization is the spin diffusion of free electrons”. Since it’s the main observation of this work, is there more experimental evidence for this attribution? For example, can the diffusion coefficient and/or spin relaxation time be varied to verify this hypothesis? In particular, would changing the sample temperature affect electron diffusion and electron spin lifetime? Does the temperature affect the synchronization effects and the spatial correlations? Or is it possible to control electron diffusion and electron spin lifetime by looking at samples with different doping concentrations -- is there data for that? I think it's an important question, since it's the essence of the physics behind the observed oscillations. Some additional data would be needed to make a convincing claim. So far, there is only some numerical modelling, but this is not very convincing when it comes to resolving the underlying mechanism, because numerical modelling will show whatever the parameters are tuned to.

“identifying spin transport as the coupling-mediating mechanism and establishing phase coherence over mesoscopic distances” in the abstract, and further “Demonstrating that diffusive spin currents can mediate both long-range phase coherence”. I don’t see any evidence in this work that there is any long-range phase spin coherence. The data does point to long-range correlations, but it doesn’t mean it’s spin coherence (i.e. phase coherence of the spin quantum state). I think some explicit experimental data would be needed to claim phase coherence. That is probably beyond current experiments, so I would suggest removing that claim. For present work, I would recommend extending numerical modelling to explore this point. For example, what if the model is limited to preserving only the longitudinal component of the electron spin (i.e. projection on the local total field) but erasing (randomising) the transverse spin component in some way, for example at each electron hop or event that leads to diffusion? If the frequency synchronization persists even when transverse spin component is randomised, then it's very unlikely to be a phase-coherence effect. In any case, whatever numerical modelling shows, it's better not to call this phase coherence until there is experimental evidence.

“We conclude that the synchronization range is 38 ± 3 μm taken between the central positions of the pump spots, or taking the spot sizes of the pump beams into account”. This is a nice dataset (Fig 3f) uncovering the spatial extent of correlated self-oscillations, but it leaves many unanswered questions. What happens at the edge of the transition between two regimes? One may expect to see spontaneous emergence or disappearance of synchronization (i.e. time-dependent transients or increased fluctuations near the phase transition boundary). Does this happen? More generally, what is the time-domain behaviour of the self-oscillation effect. What is the 0 time point on the horizontal axis of Fig. 3a – how does it relate to when the pump is switched on? How long does it take for oscillations to emerge in case of one pump beam switched on at time 0, and how does it depend on the state of the sample before pumping? How long does it take for a new steady state to emerge if there is only one pump initially and the second (spatially offset) pump is switched on at timepoint 0? The way the data is presented, makes it look like oscillations are infinite in time, with no beginning and no end. But surely there must be some initial transients for the system to enter the self-oscillation regime. Are these transients taken into account when carrying out FFT and autocorrelations. This is related to my general comment about providing adequate level of in-depth data and analysis, and I think the points raised above are well within experimental reach.

I am wondering what controls the actual period of self-oscillations? There was a comment in one of the earlier papers from the authors suggesting that the exact behaviour of the strongly coupled spin system is quite complicated. Perhaps this is true, but there must be some way to predict at least the order of magnitude of the fundamental harmonic. The system of differential equations (1 and 2) is not that complicated. It seems that there are only four independent parameters (D_s,*T_s, T_n, ^a tensor, and Hanle half width) – surely the characteristic time would be some combination of these? Have the authors tried dimensional analysis to find what controls the fundamental period? Alternatively, is it possible to find some empirical relation between the input parameters and the fundamental frequency? It looks like fitting was done, so there would be numerical modelling data for a range of input parameters. Some basic estimation of the oscillation period is the same as asking what the inherent nature of the oscillations is. Intuitively, self-oscillation is usually related to some transformation of energy between different forms (like in a pendulum or LC circuit), with some harmonics arising from nonlinearity. But it is still not very clear what is going on here (even after I read refs 28, 29). For example, what are the physical states at the minima and maxima of Faraday rotation? Once again, I think it’s an important question related to in-depth explanation of the underlying physics. It’s fine when the origin and basic properties of the oscillations are a mystery in the initial report. But if these basic properties remain a mystery in paper number 3 on the same subject, it looks somewhat strange.

“Electron spin diffusion: Spin-polarized free carriers created in one spot diffuse into the other, affecting the donor-bound electrons by the spin exchange coupling” What is the fundamental difference between hopping and diffusion of electrons in mechanisms 2 and 3? Are they not the same thing? Does the electron spin diffusion happen through electrons hopping, or is there some other mechanism?

“The first step of our study is to establish robust auto-oscillations of the electron-nuclear spin system.” That raises a question: electron-nuclear spin system of what? It wasn’t that easy to find the answer. I think the authors need to give a brief description of the sample in the introduction and also state the material system in the abstract. Moreover, it is impossible to understand what the physical setting is even after referring to Supplementary. The supplementary of this work largely refers to previous publication. I think it’s a bad practice if the reader needs to read other papers just to understand what this one is about – I found it difficult to follow the story due to these missing details. This is especially problematic because the referenced paper [28] does not seem to be an Open Access, while this submission is for an Open Access journal. If it’s the same sample, one may think that just copying the Supplementary text, with any necessary modifications, would do the job.

Equation 2 ignores nuclear quadrupolar interactions. For Indium in GaAs, there should be a large localized strain around each In atom, causing few-MHz range nuclear spin frequencies. This is larger than the hyperfine (Knight) shifts, and larger than nuclear Zeeman (at mT range fields quoted for this work), making quadrupolar dominant. Moreover, since it’s a thin InGaAs epilayer on a presumably thick substrate (what is the thickness by the way?), the epilayer should comply to the lattice constant of the substrate through biaxial-type lattice strain with principal axis along the growth direction. This type of strain would give a more uniform (as opposed to atomic-scale disorder) component of quadrupolar shift. Since the Bohr radius is quite large, the electrons should “feel” only the averaged uniform quadrupolar shift. So all electrons will have a common quadrupolar quantization axis, close to the growth axis. In any case, quadrupolar should be the dominant term, and ^a*S is a small perturbation. Equation 2 suggests the opposite assumption, but there is no justification. Unless only the +/-1/2 nuclear projection states participate in auto-oscillations, but in that case, what is the role of indium at all? That all seems illogical, and also raises a question whether the match between numerical model and experiment is just coincidental. The quadrupolar term was included in previous publications [Ref 28] with an assumption of isotropic distribution of quadrupolar axes with B_Q=a_N*S. But there was no real justification for that assumption, and, as mentioned above, I would say it’s incorrect. It looks more like that assumption B_Q=a_N*S (in Ref 28) or dropping the quadrupolar term altogether without explanation (in this manuscript) is convenient for getting the desired result, rather than explaining the physics. I think these inconsistencies should be resolved.

Some technical comments:

“The tensor has been simplified in the lowest order approximation (for details, see Methods section, Ref. [28], and the

corresponding Supplementary section 5).” Can the authors just spell out what the tensor “a” is? Presumably it’s just a 3x3 matrix, so why sending the reader to Supplementary of a paper in a different journal?

“The model suggests that ˆaS is a linear function of S.” Not clear what the authors are trying to say. Does it mean that it’s an eigen-value problem for S? Otherwise, this sentence looks like an infinite-recursion definition, which doesn’t make sense for a physical vector S. Clarification needed.

“Finally, the intrinsic variation of the semiconductor sample, related to fluctuations in donor density and indium incorporation, is expected to result in variations of the observed oscillation frequencies.” Is it an expectation, or has this been observed? This is related to one of the points above, and the question of verifying the primary hypothesis.

Reviewer #2

(Remarks to the Author)
This paper describes experiments and observations of synchronization between two electron/nuclear oscillators in the presence of a CW optical pump. The main claim centers around the observation of “non-local” synchronization. In my reading, the authors have gone to some effort to "sell" this work. However, the results are indeed interesting.

Here are my comments/questions:
1. I question the use of the term “non-local” here. In my understanding, nothing is travelling faster than the speed of light.
2. The authors argue that because of spin diffusion, the large flat-top pump produces synchronization. Is there a smallest spot size for which this effect is no longer relevant? I would imagine this smallest spot size might correspond to the “size” of the electrons, but I could be wrong. If this assumption is correct, then is spin diffusion required to explain the oscillations observed with a single, small pump spot? (Here I am trying to find for myself the precise scientific advance of this paper compared to the previous work by the authors [Nat. Phys., 20, 631–636 (2024)]).
3. It seems to me that the authors are not, strictly speaking, able to measure the oscillators independently. Instead, they conduct a global measurement and infer the properties of the two oscillators from these measurements. Is it possible that the single probe spot, instead of any synchronization, could explain these results? I ask because Fig. 2 and Fig. 4f seem to indicate that making the pump beam more homogeneous increases the level of “synchronization,” so can the same thing happen with the probe?
4. In the supplement, the authors mention “frequency pulling.” How do the authors rule out this as an explanation for the observations? Or is this related to synchronization?
5. It would be helpful for the authors to more explicitly address in the main text other potential hypotheses for their data and how they can rule them out. For example, the supplement discusses the possible idea of cross illumination by the pump spots. It would be good for some discussion of this to appear in the main text. A related (minor) point is that the hypothesis comes at the end of the paper, so one needs to go back over the paper to understand the significance of the various measurements.

Version 1:

Reviewer comments:

Reviewer #1

(Remarks to the Author)
The authors have provided detailed responses, supported by reports of new experimental data. I have no further comments and support publication in Nature Communications.

Just one comment on the following: “This local strain leads to quadrupolar splitting of nuclear spin levels for all nuclei with spin > 1/2". Is it meant to be ‘nuclear states with absolute value of spin projection >1/2’? Otherwise looks confusing because all nuclear spins are either 3/2 or 9/2 anyway.

Reviewer #2

(Remarks to the Author)
The authors have responded appropriately to the comments and have notably performed additional experiments to support their claims.

Following up on comment #2 from Reviewer #1, there are still many instances of the term coherent and its variants. These should be removed.

I would be happy for this paper to appear in Nature Communications, though I would not argue strongly for it. The reason is that some kind of collective synchronization mechanism seems required to explain, and is thus perhaps implicit in, the previous work of the authors [Nat. Phys. 20, 631–636 (2024)]. To quote the authors, the present work “clarifies their [the oscillators’] spatially collective character and identifies spin diffusion as the mechanism enabling non-local synchronization between local oscillatory subsystems” (quote from page 11 of the rebuttal). In this sense, the present paper reveals the

synchronization mechanism that was likely present and required to explain the earlier work.

Open Access This Peer Review File is licensed under a Creative Commons Attribution 4.0 International License, which permits use, sharing, adaptation, distribution and reproduction in any medium or format, as long as you give appropriate credit to the original author(s) and the source, provide a link to the Creative Commons license, and indicate if changes were made.
In cases where reviewers are anonymous, credit should be given to 'Anonymous Referee' and the source.
The images or other third party material in this Peer Review File are included in the article's Creative Commons license, unless indicated otherwise in a credit line to the material. If material is not included in the article's Creative Commons license and your intended use is not permitted by statutory regulation or exceeds the permitted use, you will need to obtain permission directly from the copyright holder.
To view a copy of this license, visit https://creativecommons.org/licenses/by/4.0/

Response to reviews of NCOMMS-25-96650-T, "Non-local synchronization of continuous time crystals in a semiconductor", by A. Greilich *et al.*

We thank the Reviewers for their constructive feedback and insightful comments. We have carefully addressed each point raised to improve the depth and clarity of the manuscript. The revisions in the main text and in the supplementary materials are highlighted in blue.

Reviewer #1

The paper reports on spatial correlation properties of self-oscillations in an optically pumped electron-nuclear spin system of a semiconductor sample. The work follows up from recent studies conducted by the same authors [Nature Phys 20 631, Nature Comm 16 2936], so there is some overlap. However, the subject of the present manuscript is quite distinct (focus on spatial correlations). The work reports nice experimental results and presents interesting physics phenomena. I broadly support publication upon adequate revision. My main general criticism is on the depth of experimental results and analysis. My feeling is that the study is somewhat superficial, reporting some experimental phenomena but without going deep enough in terms of understanding the underlying mechanisms. Some lack of details in understanding made sense for earlier reports on the subject [Nature Phys 20 631], but as research progress is made, one would expect to seem a more comprehensive study. In other words, it's fine when the initial report discovering the self-oscillation effect leaves many details in the "yet to be understood" category, but one would expect more mature analysis for a third publication on the same experimental effect. There are also some claims, which I think are not properly supported by the data (e.g. phase spin coherence).

Reply: We thank the reviewer for the positive evaluation of our experimental results and for recognizing the interesting physics revealed in this work. In our earlier publication (Nat. Phys. 20, 631) we reported the discovery of auto oscillations in an optically pumped electron–nuclear spin system and focused primarily on establishing the experimental effect itself. A detailed microscopic understanding was intentionally left for subsequent studies. In the following work (Nat. Commun. 16, 2936) we addressed one of the key aspects of this phenomenon, namely synchronization to an external drive, demonstrating a range of nonlinear dynamical effects including Arnold tongues, devil's staircase behavior, and locking at different winding numbers.

In the present manuscript, we investigate another important physical aspect of the same system, the internal mutual synchronization of a large ensemble of spatially distributed local oscillators. Our measurements of spatial correlations allow us to identify the dominant mechanism underlying this collective behavior, which we attribute to electron spin diffusion that mediates coupling between different regions of the sample.

We agree with the reviewer that developing a fully microscopic description of the coupled electron–nuclear spin dynamics is an important goal. Such a

theory is currently under development. In the present work, however, we focus on establishing the key experimental signatures of spatial synchronization and providing a physically motivated interpretation of the mechanism responsible for it. Furthermore, in the revised version, we have extended the presented data set; see details below.

The details are outlined below. Most of the comments below are linked to parts of the manuscript which are quoted as "":

1) "based on the experimentally observed synchronization range of ~ 40 μm, we can suggest that the primary mechanism responsible for the synchronization is the spin diffusion of free electrons". Since it's the main observation of this work, is there more experimental evidence for this attribution? For example, can the diffusion coefficient and/or spin relaxation time be varied to verify this hypothesis? In particular, would changing the sample temperature affect electron diffusion and electron spin lifetime? Does the temperature affect the synchronization effects and the spatial correlations? Or is it possible to control electron diffusion and electron spin lifetime by looking at samples with different doping concentrations - is there data for that? I think it's an important question, since it's the essence of the physics behind the observed oscillations. Some additional data would be needed to make a convincing claim. So far, there is only some numerical modeling, but this is not very convincing when it comes to resolving the underlying mechanism, because numerical modeling will show whatever the parameters are tuned to.

Reply: We agree that establishing the physical mechanism is crucial. To address this, we have performed additional measurements at different temperatures (6 K and 15 K) to investigate the temperature dependence of the synchronization range. We limited the upper range to 15 K because, as demonstrated in our previous work [Nat. Phys. 20, 631 (Supplementary Materials)], the auto-oscillations vanish above approximately 17 K. Interestingly, we found no significant difference in the synchronization range between 6 K and 15 K. This observation suggests that while the auto-oscillations themselves are highly sensitive to temperature, the underlying coupling mechanism remains relatively robust within this temperature window.

Furthermore, we have now tested the dependence of the synchronization range on the carrier concentration by comparing our standard sample ($n_e \approx 39 \times 10^{15}$ cm^{-3}) with a lower-density structure ($n_e \approx 0.5 \times 10^{15}$ cm^{-3}). While we were able to discover auto-oscillations at this lower concentration, the effective nonlocal coupling range was significantly reduced. In the low-density sample, the measured synchronization range between central positions of pump spots shifts to only about 35 μm, even with substantially larger pump diameters (34 μm and 50 μm) used to maintain sufficient signal-to-noise. At this separation, the $1/e^2$ excitation contours still overlap by approximately 7 μm. This stands in stark contrast to the high-density sample, where synchronization persists over a finite beam separation of about 20 μm between the contours measured at the intensity levels of $1/e^2$ of the maximum intensity.

This comparison demonstrates a strong reduction of the effective nonlocal coupling range at lower densities, identifying carrier-mediated spin transport as the primary coupling agent. The figure demonstrating these measurements has been added to the Supplementary section 5, and a corresponding discussion is now included in the main text.

2) “identifying spin transport as the coupling-mediating mechanism and establishing phase coherence over mesoscopic distances” in the abstract, and further “Demonstrating that diffusive spin currents can mediate both long-range phase coherence”. I don’t see any evidence in this work that there is any long-range phase spin coherence. The data does point to long-range correlations, but it doesn’t mean it’s spin coherence (i.e. phase coherence of the spin quantum state). I think some explicit experimental data would be needed to claim phase coherence. That is probably beyond current experiments, so I would suggest removing that claim. For present work, I would recommend extending numerical modelling to explore this point. For example, what if the model is limited to preserving only the longitudinal component of the electron spin (i.e. projection on the local total field) but erasing (randomising) the transverse spin component in some way, for example at each electron hop or event that leads to diffusion? If the frequency synchronization persists even when transverse spin component is randomised, then it’s very unlikely to be a phase-coherence effect. In any case, whatever numerical modelling shows, it’s better not to call this phase coherence until there is experimental evidence.

Reply: We appreciate this distinction. Our data demonstrates phase synchronization between two distant oscillating volumes. To avoid confusion with quantum-state coherence (T_2), we have replaced the term "phase coherence" with "long-range phase synchronization" or "correlated phase dynamics" throughout the manuscript and abstract.

We appreciate the reviewer’s suggestion regarding additional numerical tests that would explicitly suppress transverse spin coherence in the model. Such extensions of the modeling could indeed provide further insight into the role of different spin components and role of nuclear spin fluctuations in the synchronization mechanism and represent an interesting direction for future work. In the present manuscript, however, the modeling is intended primarily to capture the effect of spin diffusion and to reproduce the experimentally observed synchronization behavior.

The corresponding passages in the manuscript have been revised accordingly.

3) “We conclude that the synchronization range is 38 ± 3 μm taken between the central positions of the pump spots, or taking the spot sizes of the pump beams into account”. This is a nice dataset (Fig 3f) uncovering the spatial extent of correlated self-oscillations, but it leaves many unanswered questions. What happens at the edge of the transition between two regimes? One may expect to see spontaneous emergence or disappearance of synchronization (i.e. time-dependent transients or increased fluctuations near the phase transition

boundary). Does this happen? More generally, what is the time-domain behaviour of the self-oscillation effect. What is the 0 time point on the horizontal axis of Fig. 3a – how does it relate to when the pump is switched on? How long does it take for oscillations to emerge in case of one pump beam switched on at time 0, and how does it depend on the state of the sample before pumping? How long does it take for a new steady state to emerge if there is only one pump initially and the second (spatially offset) pump is switched on at timepoint 0? The way the data is presented, makes it look like oscillations are infinite in time, with no beginning and no end. But surely there must be some initial transients for the system to enter the self-oscillation regime. Are these transients taken into account when carrying out FFT and autocorrelations. This is related to my general comment about providing adequate level of in-depth data and analysis, and I think the points raised above are well within experimental reach.

Reply: We appreciate the reviewer's interest in the transient dynamics. However, as synchronization is a steady-state property, transient dynamics do not affect our conclusions; accordingly, all experimental measurements and numerical simulations in the present study are performed in the steady-state regime. As established in our previous studies (Nat. Phys. 20, 631 and Nat. Comm. 16, 2936), the initial transients following the switch-on of the pump beams take approximately 5 minutes to die out. All data presented in this manuscript, including the FFTs, were acquired strictly after this initial 5-minute period, ensuring that the system had reached a stable, steady-state synchronization. While investigating the transient behavior, the spontaneous emergence of synchronization, and dynamics near the transition boundary represents a highly interesting topic, it involves complex, time-dependent processes that are beyond the scope of the present work. Fully characterizing and understanding this transient behavior would require a separate, dedicated experimental and theoretical study, which we consider an exciting direction for future research.

4) I am wondering what controls the actual period of self-oscillations? There was a comment in one of the earlier papers from the authors suggesting that the exact behaviour of the strongly coupled spin system is quite complicated. Perhaps this is true, but there must be some way to predict at least the order of magnitude of the fundamental harmonic. The system of differential equations (1 and 2) is not that complicated. It seems that there are only four independent parameters (D_s, T_s, T_n, a tensor, and Hanle half width) – surely the characteristic time would be some combination of these? Have the authors tried dimensional analysis to find what controls the fundamental period? Alternatively, is it possible to find some empirical relation between the input parameters and the fundamental frequency? It looks like fitting was done, so there would be numerical modelling data for a range of input parameters. Some basic estimation of the oscillation period is the same as asking what the inherent nature of the oscillations is. Intuitively, self-oscillation is usually related to some transformation of energy between different forms (like in a pendulum or LC circuit), with some harmonics arising from nonlinearity. But it is still not very clear what is

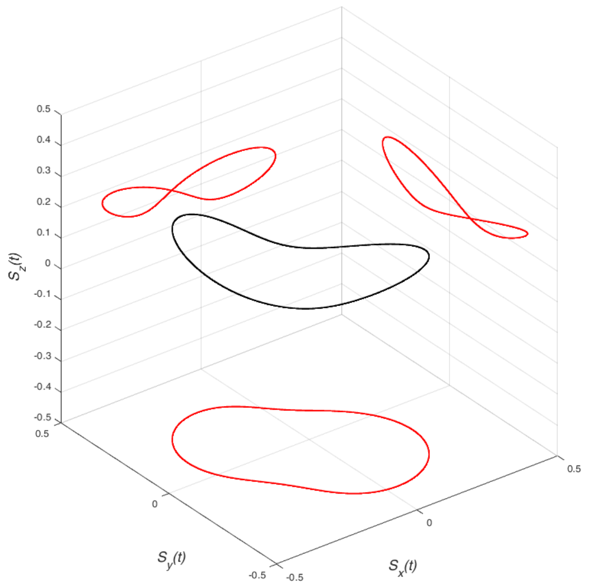

Figure 1: Black line represents a calculated evolution of all spin components. Red lines are projections on corresponding planes. The Faraday rotation method used in the experiment is only sensitive to the z-component of the spin.

going on here (even after I read refs 28, 29). For example, what are the physical states at the minima and maxima of Faraday rotation? Once again, I think it's an important question related to in-depth explanation of the underlying physics. It's fine when the origin and basic properties of the oscillations are a mystery in the initial report. But if these basic properties remain a mystery in paper number 3 on the same subject, it looks somewhat strange.

Reply: In the framework of Eqs. (1) and (2), the oscillation period is controlled by the interplay between the fast electron-spin response and the slow nuclear-spin dynamics. Since the electron spin polarization adjusts on the timescale $T_{\rm s}$, which is much shorter than the nuclear relaxation time $T_{\rm N}$, the electron subsystem effectively follows the nuclear field quasi-instantaneously. As a result, the nuclear-spin dynamics provides the natural timescale of the auto-oscillatory regime, and the fundamental oscillation frequency is expected to be of the order of $T_{\rm N}^{-1}$. At the same time, we note that for a nonlinear feedback system such as Eqs. (1) and (2), dimensional analysis alone cannot determine the oscillation frequency quantitatively. The actual period of the limit cycle is set by the nonlinear dynamics of the coupled electron-nuclear spin

system and generally requires linear stability analysis or numerical calculations. Physically, the self-oscillations arise from nonlinear feedback between the electron and nuclear spin subsystems: the Overhauser field modifies the electron spin polarization, which in turn affects the dynamic nuclear polarization with a delay determined by T_{N}. This feedback loop leads to a nonequilibrium limit cycle, which is detected experimentally by oscillations in the Faraday rotation signal. Additionally, we present a simulated figure for all spin components (see the black line in Fig. 1), which shows the 3D limit-cycle curve. The oscillations that we detect are limited to the z-projection of the system moving along that curve, measured by the Faraday rotation. So, the minima and maxima of the time series observed in the experiments are simply the points on the limit cycle, representing the evolution of the system.

5) “Electron spin diffusion: Spin-polarized free carriers created in one spot diffuse into the other, affecting the donor-bound electrons by the spin exchange coupling” What is the fundamental difference between hopping and diffusion of electrons in mechanisms 2 and 3? Are they not the same thing? Does the electron spin diffusion happen through electrons hopping, or is there some other mechanism?

Reply: We thank the reviewer for raising this important question. The two mechanisms discussed in the manuscript correspond to fundamentally different physical processes. In the hopping mechanism, the donor-bound electron itself moves between donor sites. This process involves charge transport through jumps of localized electrons and therefore requires actual electron transfer between donors. Based on our estimates, the characteristic hopping length is about $3.5\,\mu$m. In contrast, the spin diffusion mechanism does not require donor-bound electrons to move between donors. Instead, it involves the transport of spin polarization mediated by free electrons. Each donor electron is localized within a Bohr radius of about 11 nm and is coupled to approximately 10^6 nuclear spins forming a single auto-oscillator. Free electrons passing through the region interact with donor-bound electrons via spin exchange, thereby transferring spin polarization (see i.e. Paget D., PRB **24**, 3776 (1981)). As a result, the experimentally relevant quantity is the average electron spin polarization at each donor. In this picture, the spin polarization carried by free electrons diffuses over a much larger characteristic length scale (about $38\,\mu$m), effectively coupling and synchronizing multiple donor-based oscillators without requiring donor-to-donor electron hopping.

6) “The first step of our study is to establish robust auto-oscillations of the electron-nuclear spin system.” That raises a question: electron-nuclear spin system of what? It wasn’t that easy to find the answer. I think the authors need to give a brief description of the sample in the introduction and also state the material system in the abstract. Moreover, it is impossible to understand what the physical setting is even after referring to Supplementary. The supplementary of this work largely refers to previous publication. I think it’s a bad practice if

the reader needs to read other papers just to understand what this one is about – I found it difficult to follow the story due to these missing details. This is especially problematic because the referenced paper [28] does not seem to be an Open Access, while this submission is for an Open Access journal. If it's the same sample, one may think that just copying the Supplementary text, with any necessary modifications, would do the job.

Reply: We thank the reviewer for pointing this out. We agree that the description of the physical system in the original manuscript was too brief and could make it difficult for readers unfamiliar with our previous work to follow the experimental setting. In the revised manuscript, we have added a concise description of the sample (an $In_{0.03}Ga_{0.97}As$ epilayer doped with Si donors) at the beginning of the results section and mentioned it in the Introduction. The system under study consists of donor-bound electrons interacting with the nuclear spin ensemble of the host lattice, which together form the electron–nuclear spin auto-oscillators investigated in this work. We also changed the formulation in the sentence mentioned by the reviewer. In addition, we have expanded the description of the sample and experimental conditions in the Supplementary Information so that the relevant details are now fully self-contained and do not require consulting Ref. [28]. We believe these additions make the physical setting of the experiment much clearer.

7) Equation 2 ignores nuclear quadrupolar interactions. For Indium in GaAs, there should be a large localized strain around each In atom, causing few-MHz range nuclear spin frequencies. This is larger than the hyperfine (Knight) shifts, and larger than nuclear Zeeman (at mT range fields quoted for this work), making quadrupolar dominant. Moreover, since it's a thin InGaAs epilayer on a presumably thick substrate (what is the thickness by the way?), the epilayer should comply to the lattice constant of the substrate through biaxial-type lattice strain with principal axis along the growth direction. This type of strain would give a more uniform (as opposed to atomic-scale disorder) component of quadrupolar shift. Since the Bohr radius is quite large, the electrons should "feel" only the averaged uniform quadrupolar shift. So all electrons will have a common quadrupolar quantization axis, close to the growth axis. In any case, quadrupolar should be the dominant term, and $a * S$ is a small perturbation. Equation 2 suggests the opposite assumption, but there is no justification. Unless only the +/-1/2 nuclear projection states participate in auto-oscillations, but in that case, what is the role of indium at all? That all seems illogical, and also raises a question whether the match between numerical model and experiment is just coincidental. The quadrupolar term was included in previous publications [Ref 28] with an assumption of isotropic distribution of quadrupolar axes with $B_Q = a_N * S$. But there was no real justification for that assumption, and, as mentioned above, I would say it's incorrect. It looks more like that assumption $B_Q = a_N * S$ (in Ref 28) or dropping the quadrupolar term altogether without explanation (in this manuscript) is convenient for getting the desired result, rather than explaining the physics. I think these inconsistencies should

be resolved.

Reply: We thank the reviewer for this important comment. We agree that quadrupolar interactions can be strong in InGaAs and may exceed both the nuclear Zeeman splitting and the Knight shift for many nuclei. However, we would like to clarify that these effects are effectively taken into account in our model through the $a\mathbf{S}$ term in Eq. (2), as discussed in detail in Ref. [38, Ch. 5].

In the regime of strong quadrupolar splitting, where it exceeds the nuclear Zeeman energy, nuclear spin states with $|M_i| > 1/2$ are quantized along the principal axis $\mathbf{n}_i$ of the local electric field gradient tensor. In this case, the dynamically polarized nuclear spin for a given site is proportional to the projection of the electron spin onto this axis,

$$\mathbf{I}_{\mathrm{av},i} \propto (\mathbf{S} \cdot \mathbf{n}_i)\mathbf{n}_i. \tag{1}$$

Accordingly, the contribution of such nuclei to the Overhauser field is

$$\mathbf{B}_{Q,i} = a_i(\mathbf{S} \cdot \mathbf{n}_i)\mathbf{n}_i, \tag{2}$$

where a_i is proportional to the hyperfine coupling constant and the electron probability density at the i-th nucleus.

Since a localized electron interacts with a large number of nuclei within the Bohr radius, each characterized by its own quadrupolar axis $\mathbf{n}_i$, the total nuclear field is obtained by averaging over this distribution:

$$\mathbf{B}_Q = \sum_i a_i(\mathbf{S} \cdot \mathbf{n}_i)\mathbf{n}_i. \tag{3}$$

Two limiting cases can be distinguished. (i) If the quadrupolar axes are effectively randomly distributed within the electron localization volume, due to dominant local strain fluctuations, this averaging yields an isotropic contribution

$$\mathbf{B}_Q = a\mathbf{S}, \tag{4}$$

even in the presence of strong quadrupolar interaction. (ii) In contrast, for a uniform biaxial strain, the distribution of axes becomes anisotropic, leading to

$$\mathbf{B}_Q = (a_p S_x,\, a_p S_y,\, a_n S_z), \tag{5}$$

with strong anisotropy in the limit $a_n \gg a_p$, which suppresses self-oscillations.

Our experimental observation of robust self-oscillations over a wide range of external conditions indicates that strong anisotropy is absent in our sample, since such oscillations would be strongly suppressed in the presence of a dominant anisotropic quadrupolar field (see i.e. Dzhioev *et al.*, PRL **99**, 037401 (2007)). This suggests that local random strain dominates over any uniform biaxial component, consistent with the relatively low indium concentration (∼3%). As a result, the effective nuclear response is close to isotropic, justifying the use of the $a\mathbf{S}$ term.

Finally, the $|M| = 1/2$ nuclear states (central transitions) are only weakly affected by quadrupolar interactions and behave similarly to nuclei without quadrupolar splitting. Their contribution to the nuclear field is aligned with the external magnetic field, yielding an additional term

$$b(\mathbf{S} \cdot \mathbf{h})\mathbf{h}, \quad \mathbf{h} = \mathbf{B}/|\mathbf{B}|. \tag{6}$$

Combining both contributions, we arrive at the effective expression used in the manuscript:

$$\mathbf{B}_N = a\mathbf{S} + b(\mathbf{S} \cdot \mathbf{h})\mathbf{h}. \tag{7}$$

Thus, rather than neglecting quadrupolar interactions, our model incorporates their effect in an averaged form appropriate for a system with strong but spatially disordered quadrupolar coupling.

The main text has been extended to clarify this point.

Some technical comments:
8) "The tensor has been simplified in the lowest order approximation (for details, see Methods section, Ref. [28], and the corresponding Supplementary section 5)." Can the authors just spell out what the tensor "a" is? Presumably it's just a 3x3 matrix, so why sending the reader to Supplementary of a paper in a different journal?

Reply: We thank the reviewer for this comment. The quantity a is the hyperfine interaction tensor describing the coupling between the electron and nuclear spins. In the general case it is a 3×3 matrix. However, for the system considered here the interaction is well approximated as isotropic, and therefore the tensor reduces to a scalar in the lowest-order approximation. This simplification is already stated in the Methods section (Details on simulation model) of the main text. To avoid possible confusion, we have clarified this point in the revised manuscript by explicitly stating that the tensor educes to a scalar in our approximation. The reference to Ref. [28] and its Supplementary Information is only intended to provide additional information.

9) "The model suggests that ^aS is a linear function of S." Not clear what the authors are trying to say. Does it mean that it's an eigen-value problem for S? Otherwise, this sentence looks like an infinite-recursion definition, which doesn't make sense for a physical vector S. Clarification needed.

Reply: We thank the reviewer for pointing this out. The reviewer is correct that the original formulation could be confusing. In our model the quantity $\hat{a}$ is a constant tensor describing the hyperfine interaction, and it does not depend on the electron spin vector $\mathbf{S}$. The expression $\hat{a}\mathbf{S}$ simply denotes the linear action of the tensor a on the vector $\mathbf{S}$.

We agree that the phrase “a linear function of S” was poorly formulated and could suggest an unintended recursive definition. To avoid this confusion, we have removed this sentence and rewritten the corresponding part of the manuscript, consistent with the clarification added in response to the previous two comments.

10) “Finally, the intrinsic variation of the semiconductor sample, related to fluctuations in donor density and indium incorporation, is expected to result in variations of the observed oscillation frequencies.” Is it an expectation, or has this been observed? This is related to one of the points above, and the question of verifying the primary hypothesis.

Reply: We thank the reviewer for their comment. It is indeed observed. When scanning the pump spot across different regions of the sample, we see frequency shifts of up to 20%. This is now explicitly stated in the text as evidence of the sample’s microscopic landscape.

Reviewer #2:

This paper describes experiments and observations of synchronization between two electron/nuclear oscillators in the presence of a CW optical pump. The main claim centers around the observation of “non-local” synchronization. In my reading, the authors have gone to some effort to "sell" this work. However, the results are indeed interesting.

Here are my comments/questions:

1) I question the use of the term “non-local” here. In my understanding, nothing is travelling faster than the speed of light.

Reply: We thank the reviewer for raising this point. In the present work, the term non-local is used in the standard sense of transport physics and spintronics, where it refers to situations in which the dynamics at a given spatial location is influenced by spin transport originating from distant regions of the sample. In our system, electron spin diffusion mediates the coupling between spatially separated self-oscillators, leading to their synchronization over distances. In this sense, the synchronization mechanism is inherently non-local. We emphasize that the term is not used in the sense of relativistic nonlocality or any violation of causality; all interactions are mediated by spin diffusion within the sample. Similar terminology is widely used in the literature on non-local spin transport and non-local spin signals in lateral spintronic devices.

2) The authors argue that because of spin diffusion, the large flat-top pump produces synchronization. Is there a smallest spot size for which this effect is no longer relevant? I would imagine this smallest spot size might correspond to the “size” of the electrons, but I could be wrong. If this assumption is correct, then

is spin diffusion required to explain the oscillations observed with a single, small pump spot? (Here I am trying to find for myself the precise scientific advance of this paper compared to the previous work by the authors [Nat. Phys., 20, 631–636 (2024)]).

Reply: We thank the reviewer for this important question. In principle, self-oscillations in a coupled electron-nuclear spin system do not require a macroscopically extended excitation spot. The minimal physical ingredient is a localized electron-spin subsystem coupled to a mesoscopic nuclear-spin ensemble. In a semiconductor, even a single localized electron typically interacts with a large number of nuclei (roughly $10^3 - 10^6$), so, in principle, the feedback mechanism responsible for self-oscillations could already exist on a very small spatial scale.

However, an ensemble of many such local electron–nuclear oscillators is generally not composed of identical units. Different localized electrons, for example, electrons bound to different donors, experience slightly different local environments and therefore have a distribution of intrinsic oscillation frequencies. Even if each local electron–nuclear subsystem were able to oscillate individually, the macroscopic signal from a large number of mutually independent oscillators would rapidly dephase and average out. Thus, the experimentally observed collective oscillation is not simply the sum of many unrelated microscopic oscillators. It requires synchronization between them, which produces a phase-coherent macroscopic response.

In this context, the relevant length scale is therefore not the "size" of the electron, but the characteristic length over which spin diffusion can couple different local oscillators. For a finite pump spot containing many local electron–nuclear subsystems, spin diffusion provides a natural mechanism to couple these subsystems and enable the formation of a measurable synchronized response.

Regarding the relation to our previous work [Nat. Phys. 20, 631–636 (2024)], we note that the pump spot used there was of conventional size and not a spatially extended flat-top profile. Therefore, the self-oscillations reported in that work were also formed in a system where many local oscillators were present within the illuminated region and where spin diffusion within this region could contribute to their mutual synchronization. However, the key advance of the present manuscript is that we directly probe this spatial synchronization mechanism. By studying spatially separated excitation regions, we demonstrate that spin diffusion mediates synchronization between distant parts of the sample. In other words, the previous work established the existence of robust self-oscillations, whereas the present work clarifies their spatially collective character and identifies spin diffusion as the mechanism enabling non-local synchronization between local oscillatory subsystems. We have clarified this point in the revised manuscript.

3) It seems to me that the authors are not, strictly speaking, able to measure the oscillators independently. Instead, they conduct a global measurement and infer the properties of the two oscillators from these measurements. Is it possible that the single probe spot, instead of any synchronization, could explain

these results? I ask because Fig. 2 and Fig. 4f seem to indicate that making the pump beam more homogeneous increases the level of "synchronization," so can the same thing happen with the probe?

Reply: We thank the reviewer for raising this point. In our experiment, the probe beam does not create or modify the spin polarization; it only detects the Faraday rotation (FR) signal. To ensure a purely non-perturbative detection, we fix the probe photon energy at $E_{pr} = 1.426$ eV, corresponding to a local maximum of the FR response. This spectral position simultaneously provides a large FR signal while keeping the optical absorption minimal, so that the probe beam does not create or modify the spin polarization and therefore cannot couple or synchronize the oscillators. We have added more experimental details in the Methods section of the revised manuscript.

More importantly, a control experiment directly rules out such probe-induced averaging. This is demonstrated in the experiment shown in Fig. 3d,e. In this configuration, the two pump spots are separated by a distance exceeding the carrier diffusion length, so that the two regions generate independent auto-oscillations. When each pump is applied individually, two distinct oscillation frequencies are observed (Fig. 3e). When both pumps are applied simultaneously, both frequencies remain clearly visible in the Fourier spectrum. If the probe signal were merely averaging the response from the two regions, these two modes would collapse into a single effective frequency. The observation of two distinct spectral peaks therefore demonstrates that the probe detects the contributions of the two oscillators without synchronizing them.

To further support our claims, we have also performed an additional test: we placed the pump-separation at the edge of the synchronization range and tested the synchronization for different probe powers. We observed that reducing the probe power by two orders of magnitude did not affect the synchronization. The figures demonstrating this have been added to the Supplementary Materials. We have clarified this point in the revised manuscript.

4) In the supplement, the authors mention "frequency pulling." How do the authors rule out this as an explanation for the observations? Or is this related to synchronization?

Reply: We thank the reviewer for this question. Frequency pulling refers to a regime of weak coupling in which the oscillation frequencies of two oscillators shift towards each other while remaining distinct. In contrast, synchronization (frequency locking) corresponds to the regime in which the oscillators evolve at exactly the same frequency with a stable phase relationship.

5) It would be helpful for the authors to more explicitly address in the main text other potential hypotheses for their data and how they can rule them out. For example, the supplement discusses the possible idea of cross illumination by the pump spots. It would be good for some discussion of this to appear in the main text. A related (minor) point is that the hypothesis comes at the end of

the paper, so one needs to go back over the paper to understand the significance of the various measurements.

Reply: We thank the reviewer for this helpful suggestion. In the original version of the manuscript, we did not discuss the cross-illumination hypothesis in the main text because our analysis followed a general strategy of distinguishing possible mechanisms based on the characteristic spatial range over which they operate. Within this framework, mechanisms such as cross illumination can be excluded since they act only locally and cannot account for the long-range spatial correlations observed in the experiment. Nevertheless, taking the reviewer's comment into account, we have now added a brief discussion of the cross-illumination mechanism in the main text to make the rationale for excluding alternative explanations more explicit.

* * * *

Again, we thank all reviewers for helping to make this a better paper.

Response to reviews of NCOMMS-25-96650A, "Non-local synchronization of continuous time crystals in a semiconductor", by A. Greilich *et al.*

We would like to thank the Reviewers for their positive evaluation of the manuscript. Please find below a point-by-point response to all comments.

Reviewer #1 (Remarks to the Author):
The authors have provided detailed responses, supported by reports of new experimental data. I have no further comments and support publication in Nature Communications.

Just one comment on the following: “This local strain leads to quadrupolar splitting of nuclear spin levels for all nuclei with spin > 1/2". Is it meant to be ‘nuclear states with absolute value of spin projection >1/2’? Otherwise looks confusing because all nuclear spins are either 3/2 or 9/2 anyway.

Reply: *We thank the Reviewer for this hint and have adjusted the sentence accordingly. It reads now: “This local strain leads to quadrupolar splitting of nuclear spin levels for all nuclear states with an absolute value of spin projection >1/2, causing the nuclear spins to align along local principal axes h_i of the electric field gradient tensor rather than along the external magnetic field.”*

Reviewer #2 (Remarks to the Author):
The authors have responded appropriately to the comments and have notably performed additional experiments to support their claims.

Following up on comment #2 from Reviewer #1, there are still many instances of the term coherent and its variants. These should be removed.

Reply: *We thank the referee for this comment. In response, we have carefully reviewed the manuscript and removed or replaced remaining instances of the term “coherent” and its variants where they could lead to ambiguity or confusion. We now use more precise terminology to describe the observed collective oscillatory dynamics.*

I would be happy for this paper to appear in Nature Communications, though I would not argue strongly for it. The reason is that some kind of collective synchronization mechanism seems required to explain, and is thus perhaps implicit in, the previous work of the authors [Nat. Phys. 20, 631–636 (2024)]. To quote the authors, the present work “clarifies their [the oscillators’] spatially collective character and identifies spin diffusion as the mechanism enabling non-local synchronization between local oscillatory subsystems” (quote from page 11 of the rebuttal). In this sense, the present paper reveals the synchronization mechanism that was likely present and required to explain the earlier work.

Reply: *We thank the referee for this thoughtful comment. We agree that the present work sheds light on why the oscillations observed in our previous study are so robust. At the same time, we would like to emphasize that the manuscript is not intended solely as a retrospective explanation of the earlier results. The previous work indicated the presence of robust oscillatory dynamics, but did not establish whether these oscillations were spatially collective, how local oscillatory regions were coupled, or what microscopic mechanism enabled synchronization over non-local distances. The present work addresses these questions directly by demonstrating synchronization between local oscillatory subsystems and by identifying spin diffusion as the physical mechanism that mediates this collective behavior. In this sense, the manuscript both clarifies the origin of the previous observations and establishes a new, experimentally supported synchronization mechanism.*
